# NOX activation in reactive astrocytes regulates astrocytic LCN2 expression and neurodegeneration

**DOI:** 10.1038/s41419-022-04831-8

**Published:** 2022-04-19

**Authors:** Ruijia Liu, Jun Wang, Yang Chen, Jenelle M. Collier, Okan Capuk, Shijie Jin, Ming Sun, Sujan K. Mondal, Theresa L. Whiteside, Donna B. Stolz, Yongjie Yang, Gulnaz Begum

**Affiliations:** 1 Department of Neurology, The First Affiliated Hospital of Harbin Medical University, Harbin, Heilongjiang China; 2 Department of Neurology, the Pittsburgh Institute for Neurodegenerative Diseases, University of Pittsburgh, Pittsburgh, PA USA; 3 Department of Neurobiology, University of Pittsburgh, Pittsburgh, PA USA; 4 Department of Neuroscience, Tufts University School of Medicine, Boston, MA 02111 USA; 5 Department of Cell Biology, University of Pittsburgh, Pittsburgh, PA USA; 6 Department of Pathology, University of Pittsburgh and UPMC Hillman Cancer Center, Pittsburgh, PA USA

**Keywords:** Cell death in the nervous system, Astrocyte

## Abstract

Reactive astrocytes (RA) secrete lipocalin-2 (LCN2) glycoprotein that regulates diverse cellular processes including cell death/survival, inflammation, iron delivery and cell differentiation. Elevated levels of LCN2 are considered as a biomarker of brain injury, however, the underlying regulatory mechanisms of its expression and release are not well understood. In this study, we investigated the role of astrocytic Na^+^/H^+^ exchanger 1 (NHE1) in regulating reactive astrocyte LCN2 secretion and neurodegeneration after stroke. Astrocyte specific deletion of *Nhe1* in *Gfap-Cre*^*ER+/*−^*;Nhe1*^*f/f*^ mice reduced astrogliosis and astrocytic LCN2 and GFAP expression, which was associated with reduced loss of NeuN^+^ and GRP78^+^ neurons in stroke brains. In vitro ischemia in astrocyte cultures triggered a significant increase of secreted LCN2 in astrocytic exosomes, which caused neuronal cell death and neurodegeneration. Inhibition of NHE1 activity during in vitro ischemia with its potent inhibitor HOE642 significantly reduced astrocytic LCN2^+^ exosome secretion. In elucidating the cellular mechanisms, we found that stroke triggered activation of NADPH oxidase (NOX)-NF-κB signaling and ROS-mediated LCN2 expression. Inhibition of astrocytic NHE1 activity attenuated NOX signaling and LCN2-mediated neuronal apoptosis and neurite degeneration. Our findings demonstrate for the first time that RA use NOX signaling to stimulate LCN2 expression and secretion. Blocking astrocytic NHE1 activity is beneficial to reduce LCN2-mediated neurotoxicity after stroke.

## Introduction

Reactive astrocytes (RA) in stroke and other neurodegenerative diseases often gain toxic functions and have negative impact on neuronal function and survival [1, 2]. For example, in response to acute ischemic stroke, RA release several proinflammatory cytokines (TNF-α, IL-6, and IFN-γ), free radicals, and matrix metalloproteinases (MMPs), which collectively leads to neuronal death, and contributes to infarct progression [3, 4]. Recent research shows that RA also increase the expression and release of LCN2 in response to infection, inflammation or injury [5, 6]. Increase in LCN2 protein level was detected in human post-mortem brain tissues of Alzheimer’s disease, Parkinson’s disease (PD), and multiple sclerosis patients [7–9]. Elevated plasma levels of LCN2 have also been detected in ischemic and hemorrhagic stroke patients and are associated with worsened clinical outcome [10] and LCN2 is currently employed as a biomarker of brain injury [11, 12]. LCN2 is shown to be involved in neuroinflammation and neuronal death in various animal brain injury models including cerebral ischemia [13, 14]. Enhanced LCN2 expression after cerebral ischemia contributes to neuronal death by promoting glial activation, neuroinflammation, and the BBB disruption [15]. LCN2 released from astrocytes induced caspase 3-mediated neuronal apoptosis in mice treated with psychotic stimulant methamphetamine (METH) [16]. Increased expression of LCN2 in RA in kainic acid-treated mice is associated with neuronal death due to induction of iron overload and oxidative stress [17].

Despite the significance of LCN2 in brain injury, the underlying regulatory mechanisms of its expression and release from astrocytes are not well understood. Endoplasmic reticulum (ER) stress has been shown to contribute to LCN2 mRNA and protein upregulation in astrocytes and renal cells [18, 19]. ROS-mediated ER stress activation in astrocytes leads to neuronal apoptosis in the METH-induced neuronal apoptosis model [16]. Several transcription factors have been identified as important regulators of LCN2 gene expression [5], including TNF-α and IL6-mediated activation of nuclear factor κB (NF-κB) and IκBζ [20]. However, how RA regulate LCN2 expression in ischemic stroke conditions remains to be established.

We recently reported that selective knockout of *Nhe1* in astrocytes reduced reactive astrocyte formation and preserved the BBB integrity via stimulating Wnt/β-catenin signaling in stroke brains [21, 22]. In this study, we found that astrocyte-specific deletion of *Nhe1* not only attenuated *Lcn2* gene and protein expression in GFAP^+^ astrocytes but also reduced stroke-induced neurodegeneration. Activation of ER stress and UPR in astrocytes did not change LCN2 protein expression, instead, RA utilized NOX signaling to stimulate LCN2 expression and secretion. NHE1 protein activity in astrocytes is involved in accelerating NOX4-NF-κB signaling pathway. Thus, targeting astrocytic NHE1 protein is beneficial to reduce LCN2-mediated neurotoxicity after ischemic stroke.

## Results

### *Nhe1* Astro-KO mice displayed enhanced resistance to neurodegeneration after stroke

**Fig. 1:**
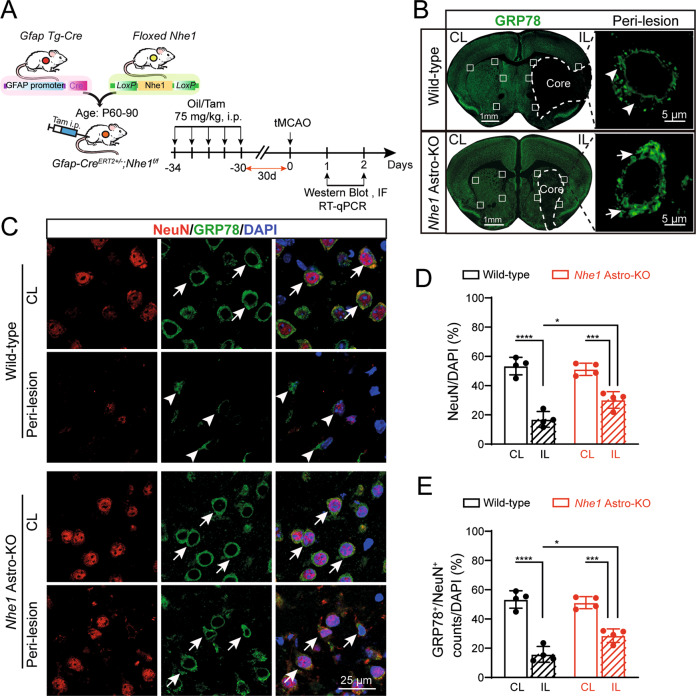
*Nhe1* Astro-KO brains displayed preserved neuronal GRP78 expression and NeuN^+^ cell counts after ischemic stroke. **A** Experimental protocol and timeline. **B** Representative immunofluorescence images of GRP78 protein expression in wild-type and *Nhe1* Astro-KO brains at 48 h Rp. High magnification images show peri nuclear GRP78 staining in peri-lesion areas indicated by white squares. **C** Representative confocal images of NeuN and GRP78 staining in neurons. **Arrows**: high GRP78 protein expression. **Arrowheads**: low GRP78 expression. **D, E** Summary data of GRP78^+^ and NeuN^+^ cells. Data are mean ± SD, *n* = 4. *p < 0.05; **p < 0.01; ***p < 0.001; ****p < 0.0001 via one-way ANOVA. CL contralateral, IL Ipsilateral hemispheres.

Astrocyte specific knockout of *Nhe1* in *Nhe1* Astro-KO mice was established in our original study (Supplemental Fig. 1 of [21]), demonstrating with immunofluorescence analysis that the lack of NHE1 protein expression in GFAP^+^ astrocytes was observed in *Nhe1* Astro-KO brains but not in the wild-type control brains. To investigate the impact of astrocyte-specific deletion of *Nhe1* on neurodegeneration, wild-type control and *Nhe1* Astro-KO mice underwent ischemic stroke (Fig. 1A). We first assessed neurodegeneration by examining loss of neuronal glucose-regulated protein 78 (GRP78), an ER chaperone protein important for ER and mitochondrial homeostasis [23], and loss of neuronal GRP78 protein is a marker for neurodegeneration [24]. As shown in Fig. 1B, wild-type brains expressed abundant levels of GRP78 in non-stroke brain regions. However, complete loss of GRP78 protein expression was detected in the ischemic core at 48 h reperfusion. Double immunofluorescence staining showed abundant expression of GRP78 in NeuN^+^ neurons in the non-stroke contralateral (CL) hemispheres (Fig. 1C). A concurrent loss of NeuN^+^/GRP78^+^ cells was detected in the peri-lesion of ipsilateral (IL) hemispheres (*p* < 0.0001). Interestingly, the stroke-induced loss of GRP78^+^/NeuN^+^ neurons was significantly reduced in the *Nhe1* Astro-KO brains, compared to the wild-type control brains (*p* = 0.0178) (Fig. 1D, E), indicating less stroke-induced neurodegeneration in *Nhe1* Astro-KO brains. These findings suggest that changes of astrocyte functions in the *Nhe1* Astro-KO attenuated stroke-induced neurodegeneration.

### Reduced astrocytic LCN2 protein expression in the *Nhe1* Astro-KO mice was associated with preservation of GRP78^+^/NeuN^+^ neurons

**Fig. 2:**
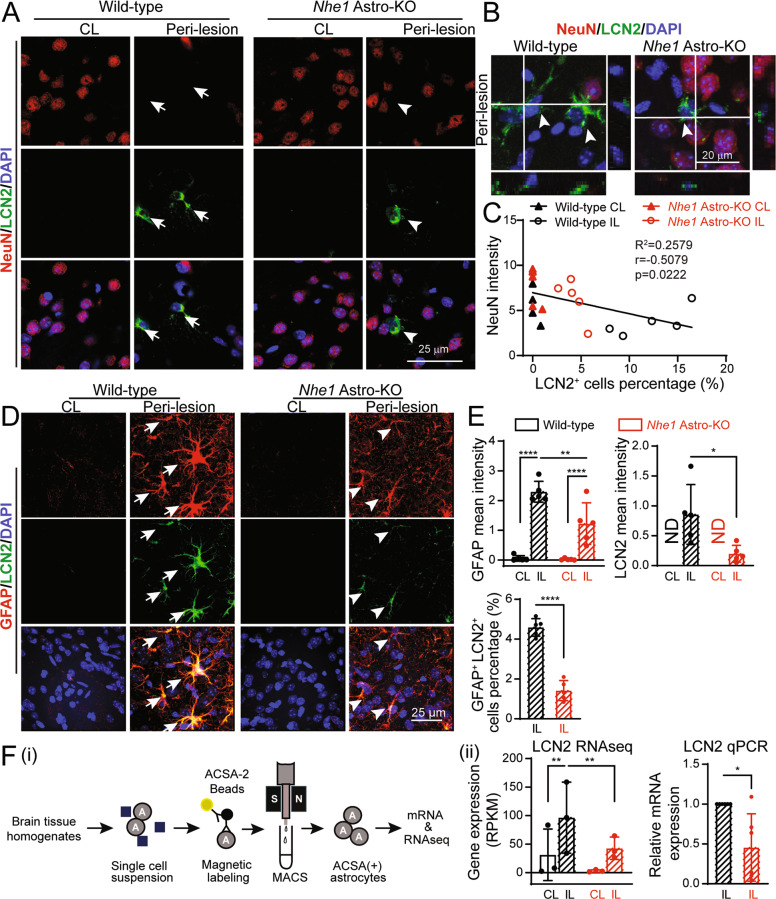
Stroke-induced LCN2 expression in RA is negatively associated with NeuN^+^ neuronal counts. **A** Representative confocal images of NeuN and LCN2 expression in astrocytes at 48 h Rp. **Arrows**: high expression. **Arrowheads:** low expression. **B** Orthogonal sections from z stack confocal images showing association of NeuN^+^ neurons with LCN2^+^ astrocytes (arrows). **C** Negative correlation between LCN2 expression and NeuN^+^ intensity in wild-type and *Nhe1* Astro-KO brains. *r* = −0.5079*, p* = 0.0222. **D** Representative confocal images of GFAP and LCN2 expression in astrocytes at 48 h Rp. **Arrows**: high expression. **Arrowheads:** low expression. **E** Summary data shows quantification of GFAP and LCN2 fluorescence signal intensity and summary data of GFAP and LCN2 double positive cells percentage. Data are mean ± SD, *n* = 5. *p < 0.05; **p < 0.01; ***p < 0.001; ****p < 0.0001 via one-way ANOVA and unpaired *t-*test. **F** (i) Cartoon illustrates isolation of astrocyte by MACS. (ii) Relative gene expression of LCN2 expressed as RPKM normalized values and RT-qPCR analysis of changes in expression of LCN2 mRNA in the astrocytes isolated from wild-type and *Nhe1* Astro-KO brains at 24 h Rp. Data are mean ± SD *n* = 6, *p < 0.05 by unpaired t-test.

In assessing RA in relation with degeneration of NeuN^+^ neurons, we found that loss of NeuN expressing neurons in the peri-lesion areas of wild-type brains was associated with reactive GFAP^+^ astrocytes expressing increased LCN2 protein (**Arrows**, Fig. 2A, B). An association between increased LCN2 protein expression and stroke-induced loss of GRP78 protein in neurons was also observed (Supplementary Fig. S1). In the ischemic peri-lesion areas of the wild-type stroke brains, RA exhibited both increased GFAP and LCN2 protein expression (Fig. 2D, E) that was not detected in neurons or microglia (Supplementary Fig. S2). In contrast, the *Nhe1* Astro-KO brains exhibited less degeneration of NeuN^+^ neurons as well as less LCN2 upregulation in GFAP^+^ RA (**Arrowheads**, Fig. 2A, B), with a negative correlation between LCN2 expression in GFAP^+^ RA and NeuN^+^ neuronal counts (Fig. 2C, Pearson coefficient of r = −0.508, *p* = 0.022). These findings suggest a possible relationship between elevated astrocytic LCN2 expression and neurodegeneration in stroke brains. To further explore this possibility, we conducted analysis of *Lcn2* mRNA expression in astrocytes isolated from wild-type and *Nhe1* Astro-KO ischemic brains by qRT-PCR and bulk RNA sequencing dataset reported recently [22]. As shown in Fig. 2F, astrocytes from IL hemispheres of wild-type brains showed a significant increase in *Lcn2* mRNA (*p* < 0.01). In contrast, *Lcn2* mRNA in astrocytes from *Nhe1* Astro-KO ischemic brains remained low (Fig. 2F). Taken together, these data strongly suggest that reduced LCN2 expression in GFAP^+^ RA in *Nhe1* Astro-KO ischemic brains may play a role in preventing neuronal loss.

### Astrocytic ER stress activation did not cause LCN2 protein upregulation

**Fig. 3:**
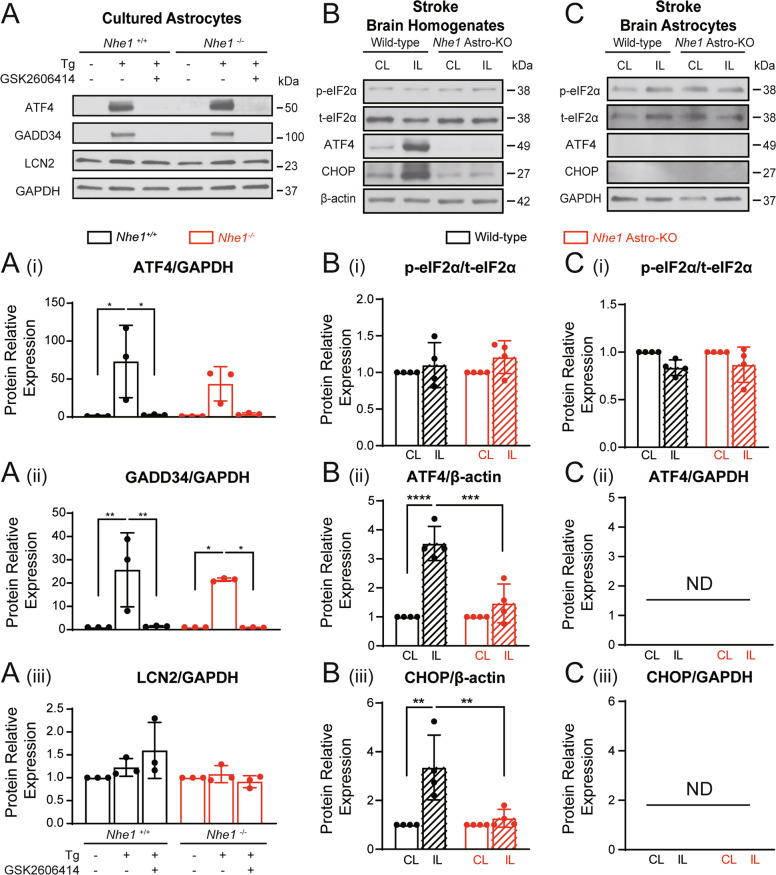
ER stress response-related protein activation in wild-type and *Nhe1* Astro-KO brains. **A** Representative immunoblots showing the expression of ER stress proteins in astrocyte cultures from wild-type (^+/+^) and global *Nhe1* KO (^−/−^) mice treated with ER stress inducer thapsigargin (Tg) 100 nM or the pathway inhibitor GSK2606414 (5 μM) for 24 h. (i-iii) Summary. Data are mean ± SD, *n* = 3. *p < 0.05; **p < 0.01 via one-way ANOVA. **B,** **C** are representative immunoblots showing the expression of ER stress proteins in brain homogenate and astrocytes isolated from wild-type and *Nhe1* Astro-KO brain at 24 h Rp. CL Contralateral, IL Ipsilateral hemisphere. (i-iii) Quantification of p-eIF2α/t-eIF2α, ATF4 and CHOP protein bands. Data are expressed as relative change (fold) of the CL controls. Data are mean ± SD*, n* = 4. *p < 0.05; ** *p* < 0.01; *** *p* < 0.001; ****p < 0.0001 via one-way ANOVA.

The mechanisms underlying LCN2 upregulation in RA are not well understood. Activation of PERK pathway, a main branch of the UPR [25] in astrocyte cultures by ER stressor thapsigargin (Tg) has been shown to increase expression of *Lcn2* mRNA [18]. To determine whether ER stress-dependent mechanisms play a role in regulating astrocytic LCN2 expression in ischemic stroke, we first tested whether Tg differently induced ER stress responses and LCN2 expression in primary astrocyte cultutres isolated from wild-type (*Nhe1*^*+/+*^) and global *Nhe1* KO (*Nhe1*^*−/−*^) mice. As shown in Fig. 3A (i) and (ii), Tg triggered significant upregulation of both ATF4 (*p* = 0.014) and GADD34 (*p* = 0.0057), two downstream proteins of PERK pathway, in *Nhe1*^+/+^ astrocyte cultutres but only GADD34 upregulation in global *Nhe1*^−/−^ astrocytes (*p* = 0.0213). These changes were blocked by PERK inhibitor GSK2606414 (Fig. 3A). However, Tg treatment did not cause any significant changes of LCN2 protein expression in either *Nhe1*^+/+^ or global *Nhe1*^−/−^ astrocytes (Fig. 3A (iii)). This was further illustrated by lack of ER stress induction in astrocytes after OGD/REOX (Supplementary Fig. S3), suggesting ER stress induction does not affect LCN2 expression in *Nhe1*^+/+^ or global *Nhe1*^−/−^ astrocytes.

We next examined whether ischemic stroke triggers PERK pathway activation differently in wild-type and *Nhe1* Astro-KO brains. Immunoblotting analysis of p-eIF2α/t-eIF2α, ATF4 and CHOP protein expression in brain homogenates (Fig. 3B) revealed that ischemic stroke triggered a significant increase in the expression of ATF4 (*p* < 0.0001) and CHOP proteins (*p* = 0.002) in the wild-type ischemic brains but not in the *Nhe1* Astro-KO ischemic brains (Fig. 3B (i) and (ii)). No significant changes of p-eIF2α/t-eIF2α expression were detected in either wild-type or *Nhe1* Astro-KO ischemic brain homogenates (Fig. 3B (i)). Considering that our immunostaining data showed that loss of ER chaperone GRP78 was mainly in NeuN^+^ neurons but not in astrocytes, we speculate that the changes of ATF4 and CHOP expression in brain homogenates mainly represent neuronal changes. To validate this, MACS-isolated astrocytes were obtained from wild-type and *Nhe1* Astro-KO brains. As shown in Fig. 3C, no changes in p-eIF2α/t-eIF2α expression were observed in wild-type or *Nhe1* Astro-KO astrocytes. The expression of PERK pathway proteins ATF4 and CHOP in astrocytes was not detectable, which is consistent with other reports in mild ER stress [26]. Our results further suggest that stroke-induced astrocytic LCN2 upregulation is likely mediated by ER stress-independent mechanisms.

### Ischemia induced increases in LCN2 secretion in astrocytic exosomes

**Fig. 4:**
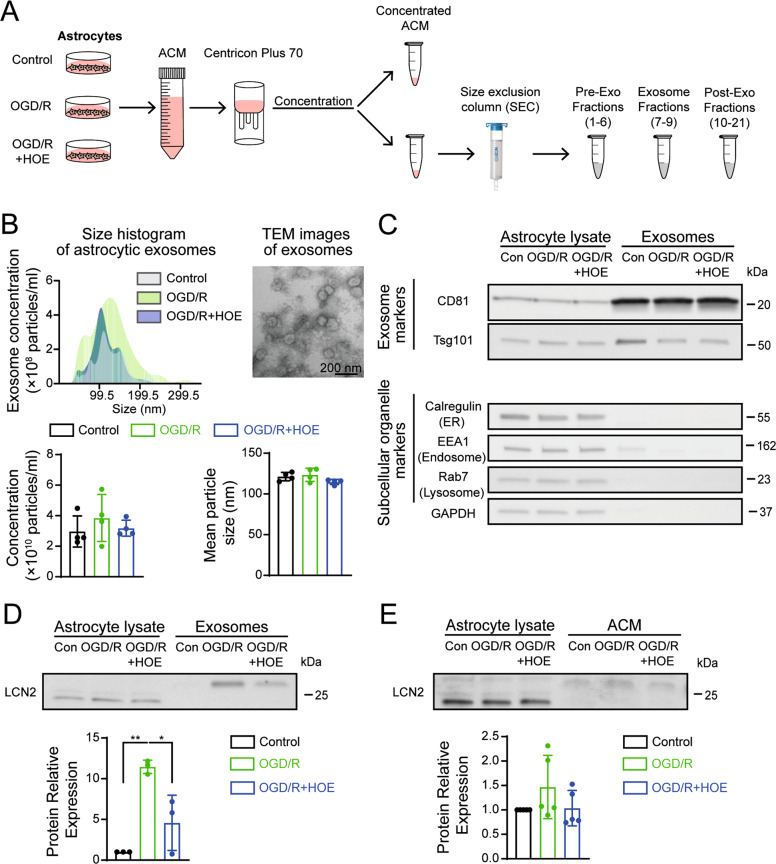
In vitro ischemia-induced differential changes in exosome LCN2 expression in primary astrocytes in presence or absence of HOE642. **A** Cartoon illustrates ACM concentration and exosomes isolation. **B** Representative size distribution of exosomes isolated from ACM of control, OGD/R and OGD/R + HOE642 treated astrocytes by NTA and representative TEM images of exosomes. Bar graph represents the quantification of exosome concentration and mean particle size. Data are mean ± SD, *n* = 4. **C** Representative immunoblots of exosome markers and other subcellular organelles specific protein markers in whole-cell lysates and purified exosome fractions. **D** Representative immunoblots of LCN2 in whole-cell lysate and exosome fractions. Bar graph represents the quantification of LCN2 band intensity. Data are mean ± SD, *n* = 3. *p < 0.05; **p < 0.01 via one-way ANOVA. **E** Representative immunoblots and quantification of LCN2 expression from astrocyte lysate and ACM isolated from control, OGD/R and OGD/R + HOE642 treated astrocytes. Data are mean ± SD, *n* = 4. *p < 0.05; **p < 0.01; ***p < 0.001 via one-way ANOVA.

RA communicate with neurons via secreting exosomes containing diverse signaling molecules [27–29]. LCN2 secreted from RA contributes to neuronal death in response to brain injury caused by intracerebral hemorrhage or exposure to antipsychotic drugs [16, 30]. We speculate that NHE1 protein in astrocytes plays a role in astrocytic LCN2 protein expression and secretion. To test this, we first investigated the changes of LCN2 protein expression and secretion in astrocytes using a well-established in vitro ischemia model (3 h oxygen/glucose deprivation (OGD)/ 24 h reoxygenation (OGD/R)) with or without the NHE1 inhibitor HOE642 (Fig. 4A). Astrocyte conditioned media (ACM) and exosomes were harvested from primary astrocyte cultures from mouse brains under either normoxic control or in vitro ischemic conditions. Exosomes were isolated from the ACM by size exclusion chromatography (SEC) column and subsequently characterized by particle size using nanoparticle tracking analysis (NTA). NTA in Fig. 4B shows that the average peak size of exosomes derived from normoxic and in vitro ischemic astrocytes were 121.4 ± 4.4 and 123.6 ± 6.8 nm, respectively. Immunoblot analysis of the exosomes from normoxic and in vitro ischemic astrocytes showed no changes in the expression of selective exosome markers, TSG 101 and CD81 (Fig. 4C). Moreover, the ER (Calregulin), endosome (EEA1) and lysosome (Rab7) markers were detected only in the astrocyte lysates, but not in exosome fractions, indicating the purity of exosomes (Fig. 4C). Compared to normoxic exosomes, a significant increase in the expression of LCN2 protein was detected in the in vitro ischemia exosomes (*p* = 0.0017; Fig. 4D). Treatment of astrocytes with HOE642 during OGD/R significantly reduced the expression of LCN2 protein in astrocytic exosomes (*p* = 0.0135; Fig. 4D). We also detected the LCN2 expression in ACM from different conditions. Immunoblot analysis revealed that in vitro ischemia moderately stimulated LCN2 protein expression in OGD/R ACM (*p* = 0.23, Fig. 4E). However, the OGD/R ACM from the astrocyte cultures treated with HOE642 did not show increased LCN2 protein expression. We speculated that the failed detection of the differences of LCN2 in ACM or cellular lysates under these conditions is due to LCN2 protein packaged into astrocytic exosomes. To assess changes of the LCN2 expression in brain exosomes, exosomes were isolated from CL and IL hemispheres of wild-type and *Nhe1* Astro-KO brains by differential ultracentrifugation [31]. As shown in Supplementary Fig. S4, stroke triggered increase in LCN2 expression (~20 and 25 kDa) in exosomes enriched fraction 2 from the IL hemispheres of wild-type brains. In contrast, low-level expression of LCN2 protein was detected in the *Nhe1* Astro-KO brain exosomes. Interestingly, a higher molecular weight LCN2 protein band (~28 kDa) was detected in exosomes isolated from cultured astrocytes, likely due to protein glycosylation of LCN2 [32]. Studies indicate that exosomes and micro vesicles are enriched in proteins with complex N-linked glycans which is required for their proper trafficking to exosomes [33]. To determine whether the different sizes of LCN2 are due to glycosylation, astrocyte cultures were incubated either with vehicle (DMSO) or N-glycosylation inhibitor tunicamycin (Tu, 2-3 µg/ml) for 24 h. As shown in the Supplementary Fig. S5, tunicamycin treatment, but not the vehicle control, resulted in increases of additional LCN2 bands (~20 kDa and 25 kDa) in both cell lysates and exosomal fractions, representing deglycosylated LCN2 proteins [34]. The prominent 28 kDa LCN2 band in exosomes was reduced by the tunicamycin treatment but not abolished, implying for the presence of additional N-glycosylation sites of LCN2 in exosomes from ACM. Together, these data suggest that RA secrete LCN2 protein packaged in the exosomes and astrocytic NHE1 protein activity is involved in LCN2 expression and secretion.

### In vitro ischemia-induced astrocytic LCN2 secretion caused neurodegeneration

**Fig. 5:**
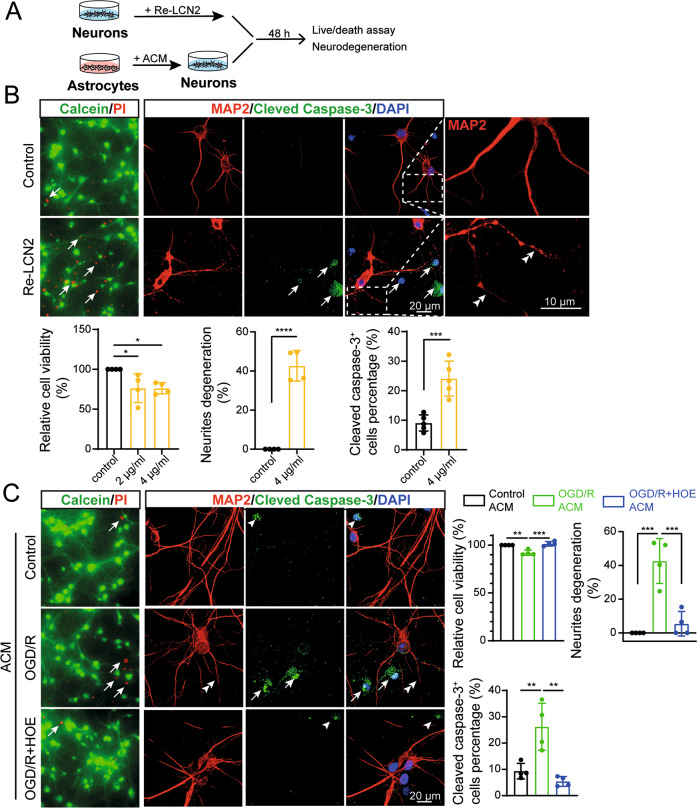
ACM from NHE1 inhibited astrocytes induced by in vitro ischemia are protective against neuronal degeneration. **A** Experimental protocol. Cartoon illustrates primary neurons treated with recombinant LCN2 (Re-LCN2) and ACM collected from different treatment groups at 24 h REOX. **B** Left panel: Representative Calcein-AM (green)/propidium iodide (red) staining of primary neurons treated with Re-LCN2 for 48 h. Right Panel: Representative confocal images of MAP2 and cleaved caspase-3 stained neurons treated with 4 μg/ml Re-LCN2 for 48 h. **Arrows**: high expression. **Arrowheads:** low expression. **Double arrowheads:** neurites degeneration. Summary data shows quantification of cell viability, percentage of neurite degeneration and cleaved caspase-3 expression. Data are mean ± SD, *n* = 4. *p < 0.05; **p < 0.01; ***p < 0.001; ****p < 0.0001 via one-way ANOVA and unpaired t-test. **C** Representative Calcein/PI staining and MAP2/cleaved caspase-3 staining of primary neurons treated with ACM of control, OGD/R and OGD/R + HOE642 treated astrocytes for 48 h. **Arrows**: high expression. **Arrowheads:** low expression. **Double arrowheads:** neurites degeneration. Summary data shows quantification of cell viability, percentage of neurite degeneration and cleaved caspase-3 expression. Data are mean ± SD, *n* = 4. *p < 0.05; **p < 0.01; ***p < 0.001 via one-way ANOVA and unpaired t-test.

To test our hypothesis that increased LCN2 expression in RA causes adjacent neuron degeneration in wild-type stroke brains, we first evaluated the effect of recombinant LCN2 (Re-LCN2) protein on neuronal viability and degeneration in cultures. Primary neurons were treated with 2 or 4 μg/ml Re-LCN2 for 48 h and neuronal viability was measured by calcein/PI staining (Fig. 5A). As shown in Fig. 5B, Re-LCN2 treatment caused a significant increase in PI^+^ neurons (**Arrows**, Fig. 5B), which failed to retain cellular calcein staining, indicating LCN2-mediated neuronal damage. We further evaluated neuronal damage by immunostaining for loss of microtubule-associated protein -2 (MAP-2) expression or increase in apoptotic marker caspase-3 (cleaved) expression. MAP-2 is localized predominantly to the neurites and perikaryon of intact neurons and loss of MAP-2 expression is indicative of neuronal damage [35]. Figure 5B revealed a significant increase in neurite blebbing and expression of the cleaved caspase-3 in the Re-LCN2-treated primary neuron cultures. These neurotoxic effects of Re-LCN2 were prevented by preincubating the Re-LCN2 protein with neutralizing LCN2 antibody (Supplementary Fig. S6A).

To further elucidate the role of NHE1 in astrocyte-derived LCN2 protein in neurodegeneration after in vitro ischemia, we harvested ACM after in vitro ischemia from primary astrocyte cultures in the presence or absence of HOE642. Subsequent exposure of primary neuronal cultures with in vitro ischemia ACM for 48 h significantly increased the number of PI^+^ dead neurons (12.5 ± 2.9%), in comparison to basal levels of PI^+^ neuronal counts (4.9 ± 2.6%) (*p* = 0.0011; Fig. 5C). Interestingly, such an increase in the number of PI^+^ dead neurons was absent in the group treated with the ACM from the HOE642-treated astrocyte cultures (3.68 ± 1.36%). Furthermore, a significant reduction in the expression of cleaved caspase 3 (20.8%, *p* = 0.0013) and degeneration of MAP-2 containing neurites (37.2%, *p* = 0.0005) was also detected in the HOE642 ACM treatment group, indicating neuroprotection (Fig. 5C). Lastly, exposure of neurons to in vitro ischemia ACM plus LCN2 neutralizing antibody (10 μg/ml) also significantly reduced cleaved caspase-3 expression and neurite degeneration (*p* = 0.0006, Supplementary Fig. S6B). Collectively, these data show that astrocyte-derived LCN2 secretion after in vitro ischemic injury induces neuronal damage. Blocking astrocytic NHE1 protein activity with HOE642 prevented LCN2-mediated neurotoxicity.

### NHE1-NOX functional cooperation in astrocytic LCN2 expression after ischemia

**Fig. 6:**
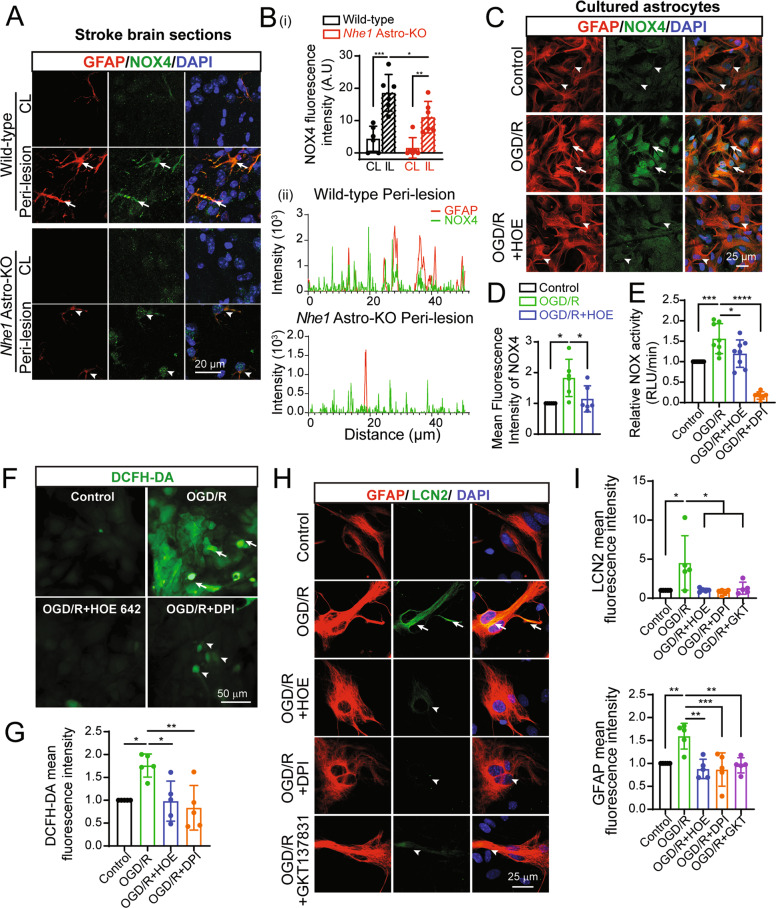
Increased NOX4 expression and NOX activity in ischemic astrocytes. **A** Representative confocal images of NOX4 and GFAP expression in astrocytes at 48 h Rp. **Arrows**: high expression. **Arrowheads:** low expression. **B** (i) Summary data shows quantification of NOX4 fluorescence signal intensity in GFAP^+^ astrocytes. (ii) Fluorescence intensity profiles of NOX4 and GFAP signals generated across the image. Note the increased overlap of the NOX4 and GFAP signals in wild-type brains. Data are mean ± SD, *n* = 6. *p < 0.05; **p < 0.01; ***p < 0.001 via one-way ANOVA. **C** Representative confocal images of NOX4 and GFAP expression in control, OGD/R and OGD/R + HOE642 treated astrocytes in cultures. **Arrows**: high expression. **Arrowheads:** low expression. **D** Summary data shows quantification of NOX4 fluorescence signal intensity in GFAP^+^ astrocytes. Data are mean ± SD, *n* = 6. *p < 0.05 via one-way ANOVA. **E** NOX activity detected using lucigenin chemiluminescence assay and expressed as relative luminescence unit (RLU)/min. Data shown are expressed as mean ± SD, *n* = 8. *p < 0.05; **p < 0.01; ***p < 0.001; ****p < 0.0001 via one-way ANOVA**. F** Representative DCFH-DA fluorescent images of normoxic, OGD/R, OGD/R + HOE642 and OGD/R + DPI treated astrocytes in cultures. **Arrows**: high expression. **Arrowheads:** low expression. **G** Summary data shows quantification of DCFH-DA fluorescence signal intensity in astrocytes. Data are mean ± SD, *n* = 5. *p < 0.05; **p < 0.01 via one-way ANOVA. **H** Representative confocal images of LCN2 and GFAP expression in control, OGD/R, OGD/R + HOE642, OGD/R + DPI and OGD/R + GKT137831 treated astrocytes in cultures. **Arrows**: high expression. **Arrowheads:** low expression. **I** Summary data shows quantification of LCN2 and GFAP fluorescence signal intensity in astrocytes. Data are mean ± SD, *n* = 5. *p < 0.05; **p < 0.01; ***p < 0.001 via one-way ANOVA.

NOX-induced oxidative stress and activation of p65 protein of the NF-κB complex play an important role in LCN2 gene induction and protein expression [36]. Since NHE1-mediated H^+^ extrusion promotes NOX-2 activation in neurons and glial cells to prevent acidic pHi-induced NOX inhibition [37, 38], we sought to determine whether increased NOX activity contributed to augmented LCN2 expression in RA after stroke. NOX4 is the major source of ROS during brain damage, and it is the predominant isoform expressed by astrocytes [39, 40]. As indicated in Fig. 6A, B, the RA in wild-type brains showed an increase in NOX4 protein expression in the ischemic peri-lesion areas (*p* = 0.0001). In contrast, reduced NOX4 expression was detected in the astrocytes from *Nhe1* Astro-KO ischemic brains (*p* = 0.04). Astrocytes subjected to in vitro ischemia also showed increase in NOX4 expression (*p* = 0.011) (Fig. 6C, D). Importantly, HOE642 treatment during in vitro ischemia reduced the NOX4 expression (*p* = 0.036). We next determined changes of the NOX activity using a superoxide-sensitive chemiluminescent probe lucigenin [41]. Under OGD/R conditions, NOX-mediated O_2_^.-^ production in astrocytes was nearly abolished by NOX inhibitor DPI (10 nM) (Fig. 6E), suggesting that the assay specifically detects NOX activity. An increase in NOX activity was detected in astrocytes subjected to in vitro ischemia (*p* = 0.001). HOE642 significantly reduced the in vitro ischemia-mediated NOX activation in astrocytes (*p* = 0.04) (Fig. 6E). We also detected ROS production in astrocytes with analysis of DCFH-DA fluorescence signals. As shown in Fig. 6F, G, DCFH-DA signals were low in normoxic astrocytes and a significant increase in DCFH-DA fluorescence was detected in astrocytes subjected to in vitro ischemia (*p* = 0.0167). HOE642 or DPI significantly reduced the in vitro ischemia-mediated ROS formation in astrocytes (HOE642; *p* = 0.0141 DPI; *p* = 0.0037). To further investigate whether increased NOX4 activity is driving LCN2 expression in astrocytes, we tested the effect of NOX4 specific inhibitor GKT137831 on the expression of LCN2 protein in astrocytes subjected to in vitro ischemia. As indicated in Fig. 6H, I, low levels of LCN2 expression were detected in astrocyte cultures under normoxic conditions. However, astrocytes subjected to in vitro ischemia showed increase in LCN2 expression (*p* = 0.0188). Inhibition of NOX activity with DPI or NOX4 specific inhibitor GKT137831 significantly reduced LCN2 expression in GFAP^+^ astrocytes (*p* = 0.0337; OGD/R vs GKT137831, *p* = 0.01; OGD/R vs DPI; Fig. 6H, I). Collectively, these data strongly suggest that NHE1 plays an important role in NOX activation and ROS production in astrocytes, which contributes to upregulation of LCN2 expression and secretion in ischemic astrocytes.

### NHE1 deficiency resulted in decreased activation of astrocytic NF-κB signaling and reduced iron accumulation in *Nhe1* Astro-KO stroke brains

**Fig. 7:**
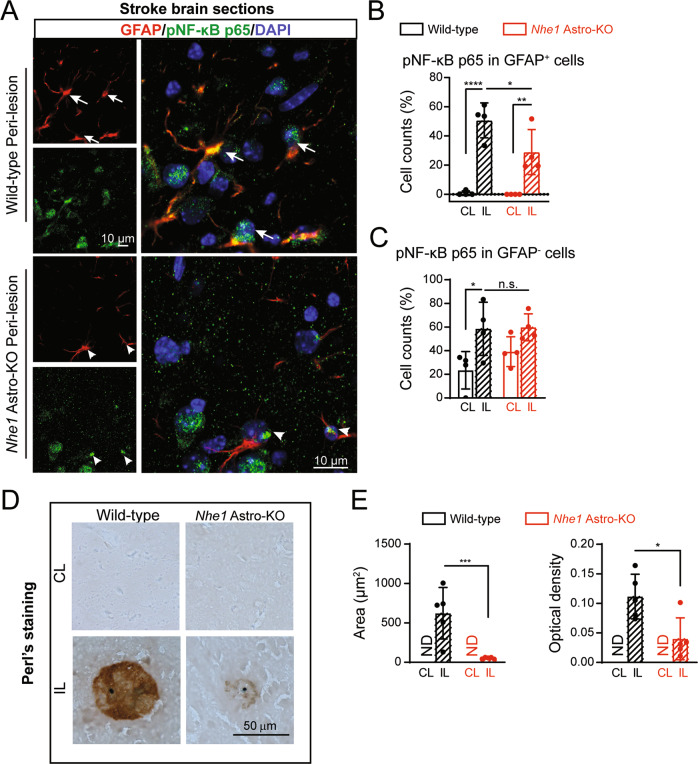
Reduced astrocytic NF-κB activation and decreased iron accumulation in astrocyte specific *Nhe1* deleted ischemic brains. **A** Representative confocal images of pNF-κB p65 and GFAP expression in wild-type and *Nhe1* Astro-KO brains at 48 h Rp. **Arrows**: high expression. **Arrowheads:** low expression. **B** Summary data shows quantification of pNF-κB p65^+^ nuclear counts in GFAP^+^ and GFAP^-^ cells. Data are mean ± SD, *n* = 5. *p < 0.05; **p < 0.01; ***p < 0.001; ****p < 0.0001 via one-way ANOVA. **C** Representative histochemical images stained for iron by enhanced Perl’s reaction. **D**, **E** Summary data shows quantification of the coverage (**D**) and optical density (**E**) of iron positive staining in the IL hemispheres of wild-type and *Nhe1* Astro-KO brains at 48 h Rp. Data are mean ± SD, *n* = 5. *p < 0.05; **p < 0.01; ***p < 0.001; ****p < 0.0001 via one-way ANOVA and unpaired t-test.

Studies show that the NOX-derived ROS can induce NF-κB signaling in astrocytes which in turn regulates the LCN2 gene expression and inflammation [36]. We therefore investigated whether lack of NHE1 in astrocytes suppresses NF-κB signaling in astrocytes after ischemic stroke, detected by changes of phospho-NF-κB p65 expression (i.e., active). We found that 50.7 ± 10.2% of all GFAP^+^ RA in the ischemic peri lesion of wild-type brains contained nuclear pNF-κB p65 signals (Fig. 7A, B). By contrast, only sparse nuclear pNF-κB p65 labeling was visible in astrocytes from *Nhe1* Astro-KO brains (29.0 ± 13.1%, *p* = 0.04, Fig. 7A, B). No significant changes were detected in the nuclear pNF-κB p65 protein expression in GFAP^-^ cells in the two groups (Fig. 7C). LCN2 contributes to an iron retentive response by promoting iron accumulation in neurons and glial cells, leading to cell damage under inflammatory conditions [30, 42]. We examined the extent of iron (Fe^3+^) accumulation in wild-type and *Nhe1* Astro-KO ischemic brains by DAB enhanced Perl’s staining. As shown in Fig. 7D, E, the CL hemispheres did not show any iron staining. However, a clear increase in iron accumulation was detected in the ischemic hemispheres. Compared to wild-type brains, *Nhe1* Astro-KO ischemic brains showed a significant reduction in coverage (*p* = 0.0003) and optical density (*p* = 0.015) of the iron-positive staining. Together, these data indicated that NHE1 protein is involved in activation of NF-κB signaling in astrocytes, which in turn regulates LCN2 secretion and LCN2-induced iron accumulation in stroke brains.

## Discussion

In response to brain injury, astrocytes undergo transformation to a reactive state characterized with elevated astrocyte proliferation and significant changes in gene expression, morphology, and cellular functions [43, 44]. Astrocytes in the reactive state can either recruit and secrete anti-inflammatory cytokines to the site of damage to contain inflammation or release proinflammatory cytokines to aggravate neuronal damage [45]. In our study, we observed that ischemic stroke-induced neurodegeneration (loss of NeuN^+^ and GRP78^+^ neurons) in the peri-lesion cortex and striatum was associated with increase in reactive astrogliosis (elevated GFAP^+^/LCN2^+^ astrocyte counts). In contrast, *Nhe1* Astro-KO brains exhibited less neurodegeneration and reduced RA. We detected significant increase in LCN2 mRNA and protein expression in RA from wild-type stroke brains but not from *Nhe1* Astro-KO stroke brains. These findings suggest that selective deletion of *Nhe1* in GFAP^+^ astrocytes modulated RA induced neurodegeneration, by regulating astrocytic LCN2 protein.

LCN2 is an acute phase glycoprotein that is rapidly produced and secreted by RA, following inflammatory and pathological stimuli [7, 46]. LCN2 contributes to neurodegenerative processes by aggravating inflammation, silencing neuroprotective pathways, and/or sensitizing neurons to cell death [13, 47]. LCN2 can regulate cellular responses by activating inflammatory pathways or by regulation of cellular iron homeostasis [48]. LCN2 binds to iron via siderophores and, this complex can transport iron into the cell by binding to LCN2 receptor, causing the neurotoxic level of intracellular iron [49, 50]. Increased secretion of LCN2 by RA contributed to the degeneration of DA neurons due to the accumulation of intracellular iron in the MPTP model of PD [7], which was decreased by an iron chelator deferoxamine [7]. How RA regulate LCN2 transcription and secretion is not well understood. Our study demonstrated that either astrocyte-specific deletion of *Nhe1* or pharmacological inhibition of NHE1 activity with HOE642 reduced the expression of LCN2 in RA (Figs. 2, 4). We also detected decreased ischemia-induced LCN2 secretion in exosomes from astrocytes with NHE1 inhibitor HOE642 (Fig. 4) or in exosomes from *Nhe1* Astro-KO stroke brains, indicating a role of NHE1 protein in the regulation of astrocytic LCN2 expression and secretion.

NOX catalyzes the production of superoxide radicals (O_2_^.-^) and generates intracellular H^+^ accumulation [37]. In colonic epithelial cells, it has been established that NOX1-derived ROS drives the expression of LCN2 via NF-κB activation and IκBζ expression [36]. Oxidative stress also induces a strong expression of LCN2 protein in colonic epithelial cells in inflammatory bowel disease [51]. NHE1 protein in astrocytes plays important roles in the regulation of pH_i_, Na^+^ concentration, and cell volume [52, 53]. Sustained NHE1 activation induced by ischemia mediates removal of intracellular H^+^, which prevents acidic pHi-dependent inhibition of NOX-mediated generation of O_2_^.-^ [37, 38]. Our study for the first time shows NHE1 in RA promotes NOX activity and ROS-mediated LCN2 upregulation. Multiple NOX isotypes (NOX2, NOX3 and NOX4) are upregulated in astrocytes upon brain injury, and our data suggest that NOX4 is the predominant isoform expressed in astrocytes. Taken together, our results indicated that increased astrocytic NOX4 activity play a role in the regulation of LCN2 transcription and expression via ROS production and NF-κB activation. Dynamic changes in intracellular pH and ROS production in RA and its role in regulating LCN2 transcription needs to be further investigated.

Exosomes are extracellular vesicles (EVs) enriched with biological molecules important in cell-cell communication [54]. Multiple studies have indicated that pharmacological inhibition of NHE activity blocks EV release [55–57] and the mechanisms are not completely understood. It has been suggested that disruption of intraluminal pH of early endosomes by NHE inhibitor can impair endosomal maturation and vesicle formation leading to reduced EV release [56]. In our study, we detected increase in LCN2^+^ exosome secretion by astrocytes subjected to in vitro ischemia. But inhibition of NHE1 activity by HOE642 reduced astrocytic LCN2^+^ exosome secretion (Fig. 4). A steady-state trans-membrane pH gradient between the endosomal lumen and cytosol is maintained by the vacuolar proton ATPases and proton leak pathways by NHEs, important for appropriate endosomal acidification [58]. We speculate that in the presence of NHE1 inhibitor HOE642, reduced NHE1-mediated H^+^ extrusion led to acidic cytosolic pH_i_ in astrocytes, which disturbs the pH gradient between the cytosol and endosomes causing intraluminal pH to be more acidic. This could affect the stability of exosomes as not all exosomal cargoes can resist conditions of low pH. Further studies are needed to investigate the precise pH_i_ regulatory mechanisms involving NHE1 protein in exosome secretions.

**Fig. 8:**
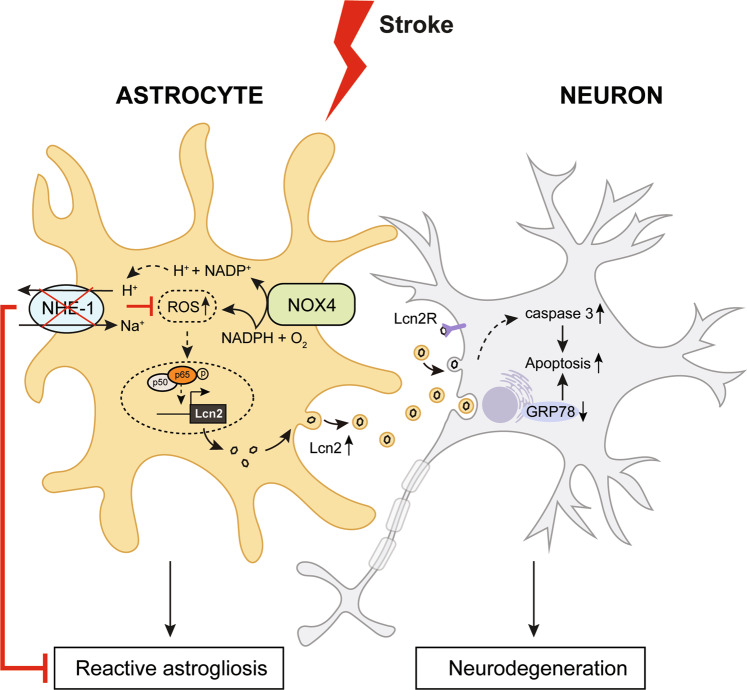
Schematic diagram illustrating NHE1 mediated mechanisms in regulation of LCN2 expression and release from stroke-induced RA. Ischemia stimulates NHE1 expression and activation in RA. Overstimulation of NHE1 activity leads to increased NOX mediated ROS production and NF-κB activation which induces LCN2 gene expression. Increased release of LCN2^+^ exosomes from RA induces neurodegeneration and neuronal apoptosis. Astrocyte specific deletion of *Nhe1* reduced LCN2 induced neuronal loss and neurodegeneration by inhibiting LCN2^+^ exosome release from RA.

In summary, our study provided appealing evidence for NHE1 in the regulation of reactive astrocyte function by modulating the expression and release of LCN2 protein after ischemic stroke. Our findings identify, for the first time, that RA use NOX-NF-κB signaling mechanisms to stimulate LCN2 expression and secretion (Fig. 8). Targeting astrocytic NHE1 is beneficial to reduce LCN2-mediated neurotoxicity after ischemic stroke.

## Methods

### Materials

Tamoxifen, DAPI (4’,6-Diamidino-2-Phenylindole, Dihydrochloride), Cariporide (HOE642), Thapsigargin, Lucigenin, NADPH, Superoxide Dismutase from bovine erythrocytes (SOD), Diphenyleneiodonium chloride (DPI), 2’-7’-dichlorofluorescein diacetate (DCFH-DA) and Centricon Plus-70 Centrifugal Filter were from Sigma-Aldrich (St. Louis, MO, USA). Amicon Ultra-4 Centrifugal Filter Unit and GSK2606414 was from Millipore (Billerica, MA, USA). iTaq™ Universal SYBR® Green, and iScript cDNA kit, were from Bio-rad laboratories (Hercules, CA, USA). GKT137831 was from Selleckchem (Houston, TX, USA). Direct-zol™ RNA MicroPrep was from Zymo Research (Irvine, CA, USA). Adult Brain Dissociation Kit, mouse and rat, Anti-ACSA-2 MicroBead Kit were from Miltenyi Biotec (Germany).

### Animals

All animal studies were approved by the University of Pittsburgh Medical Center Institutional Animal Care and Use Committee, which adhere to the National Institutes of Health Guide for the Care and Use of Laboratory Animals are reported in accordance with the Animal Research Reporting In Vivo Experiments (ARRIVE) guidelines [59]. All efforts were made to minimize animal suffering and the number of animals used.

*Nhe1*^*f/f*^ mice and *Gfap-Cre*^*ER+/−*^*;Nhe1*^*f/f*^ mice were established as described previously [21]. Astrocyte specific deletion of *Nhe1* in *Gfap-Cre*^*ER+/−*^*;Nhe1*^*f/f*^ mice (*Nhe1* Astro-KO) was induced by administration of tamoxifen (Tam, 75 mg/kg, i.p.) daily for five consecutive days in both male and female mice at postnatal days 60 to 90 (P60-90) as previously established [21] (Fig. 1A). Tamoxifen-treated *Gfap-Cre*^*ER−/−*^*;Nhe1*^*f/f*^ mice served as wild-type controls (Fig. 1A). At 30-day postinjection after clearance of Tam effects, mice underwent stroke procedures. PCR genotyping analysis was performed with genomic DNA of tail biopsies [21].

### Focal ischemic stroke model

Transient focal cerebral ischemia was induced by intraluminal occlusion of the left middle cerebral artery (MCA), as previously described [60]. Briefly, under 1.5% isoflurane, the left common carotid artery (CCA) was exposed via a midline pre-tracheal incision and the external carotid artery (ECA) and the CCA were ligated. To occlude the MCA, a rubber silicon-coated monofilament suture (size 6–0, diameter 0.09–0.11 mm, length 13 mm; diameter with coating 0.21 ± 0.02 mm; coating length 5 mm) was inserted into the ECA and advanced along the internal carotid artery 8–9 mm from the bifurcation of the carotid artery. For reperfusion, the suture was gently withdrawn 60 min after occlusion. Cranial and body temperatures were monitored with tympanic membrane and rectal temperature probes and maintained at 36.5 ± 0.5 ^o^C throughout the experiment by a heating blanket.

### Immunofluorescence staining and image analysis

#### Mouse brain sections

Mice were anesthetized and transcardially perfused as previously described [21]. Coronal brains sections (25 µm, at +1.1 mm bregma) were washed with PBS and incubated with blocking solution (10% normal goat serum (NGS), 5% BSA and 0.5% Triton X-100 in 0.01 M PBS) for 1 h at room temperature (RT) followed by incubation with primary antibodies (Supplementary Table 1). All the primary antibodies were diluted in the blocking solution containing 3% NGS and 0.3% Triton X-100 in PBS and incubated with brain sections overnight at 4 °C. On the following day, the sections or cultured cells were washed with PBS and then incubated with respective secondary Alexa 488/546 conjugated IgGs (1:200, Thermo scientific Life Technologies Corporation). DAPI (1:1000 in blocking solution) was used to stain the nucleus. For negative controls, brain sections were stained with the secondary antibody only. Fluorescent images were captured with either Olympus IX81 inverted microscope with a FV1000 laser scanning confocal system or NIKON A1R confocal microscope using a 40x, 60x or 100x, oil-immersion objective and 1024 × 1024 pixel resolution (0.103 μm/pixel).

#### Neuronal or astrocyte cultures

Cultutred cells grown on coverslips were fixed in 4% paraformaldehyde in PBS for 15 min. After rinsing, cells were incubated with a blocking solution (10% normal goat serum (NGS), 5% BSA and 0.5% Triton X-100 in 0.01 M PBS) for 1 h at RT followed by incubation with primary antibodies overnight at 4 °C. (Supplementary Table 1). After rinsing in PBS, cells were incubated with appropriate goat Alexa fluor 546/488 secondary antibodies (1:200, Thermo scientific Life Technologies Corporation) for 1 h at RT. Fluorescence images were captured with NIKON A1R confocal microscope using a 40x or 60x oil-immersion objective. For negative controls, cultures were stained with secondary antibodies only (Supplementary Fig. S7). Fluorescence images were quantified with either NIS elements (NIKON) or Fiji (NIH) software.

### Immunoblotting

Brain tissue, isolated astrocytes, astrocyte cultures, astrocyte conditioned media (ACM) or exosomes were homogenized in RIPA lysis buffer (Thermo Scientific, Rockford, IL, USA) containing protease and phosphatase inhibitor mixture (Roche) and centrifuged at 4 °C for 30 min at 14,000 × *g*. The supernatant was transferred to a fresh tube and concentration was determined by BCA kit (Thermo Scientific). An equal amount of proteins were separated by 4–15% SDS-PAGE and electrotransferred onto a PVDF membrane as described before [61]. For exosomes, equal volume of protein was separated using 4–15% SDS-PAGE. After blocking, the membrane was incubated at 4 °C overnight with primary antibodies (Supplementary Table 1). After 1 h incubation at room temperature with an appropriate secondary antibody conjugated with horseradish peroxidase (anti-rabbit antibody or anti-mouse antibody, 1:1000–1:2000), protein bands were visualized using enhanced chemiluminescence agents. The densities of bands were measured with ImageJ. Changes of target protein expression were first normalized with internal loading control protein expression.

### Isolation of astrocyte from mouse stroke brains by magnetic-activated cell sorting (MACS)

Single-cell suspension of astrocytes from the contralateral (CL) and ipsilateral (IL) hemispheres from wild-type and *Nhe1* Astro-KO brains were prepared using Adult Brain Dissociation Kit (Miltenyi Biotec, Germany) following manufacturers’ protocol. At 24 h post-tMCAO, brains were removed and placed in d-PBS (ice-cold, pH 7.2). Cortical and striatum tissues were rapidly dissected on ice and separated in enzyme mixture with gentleMACS Octo Dissociator for 30 min at 37 °C. Digested tissues were dissociated into single-cell suspension by filtration through 70 μm MACS SmartStrainer and a series of centrifugation steps as per manufacturers protocol. A total of ∼2 × 10^6^ cells were used for isolation of astrocytes by magnetic bead separation using anti-ASCA-2 microbead kit. The cell suspension was applied to the MS column, which was washed with 1−2 ml of PBS and the eluted fraction was pelleted at 300 × g for 10 min. The pelleted cells were further processed either for immunoblotting or RT-PCR analysis.

### RT-qPCR analysis

RNA of MACS-isolated astrocytes was extracted using the Direct-zol™ RNA MicroPrep kit (Zymo Research, R2060). RNA quantification was carried out by measuring absorbance with spectrophotometer ND-1000 (NanoDrop). Reverse transcription was performed using the iScript Reverse Transcription Supermix (Bio-Rad, 1708840) according to the manufacturer’s protocol. All RNA isolated from cell pellets was converted into cDNA. Quantitative RT-PCR was performed using iTaq Universal SYBR Green Supermix (Bio-Rad, 172-5120) on a CFX 96 Touch Real-Time PCR Detection System. All relative gene expression analyses were performed using the 2 − ΔΔCt method in a minimum of four animals per group with duplicate reactions for each gene evaluated. Following primer sequences were used as follows.

**Table:** 

Gene	Forward (5’ →3’)	Reverse (5’ →3’)
GAPDH	AACTTTGGCATTGTGGAAGG	ACACATTGGGGGTAGGAACA
LCN2	TGGCCCTGAGTGTCATGTG	CTCTTGTAGCTCATAGATGGTGC

### Primary cortical astrocyte or neuronal cultures

Dissociated cortical astrocyte cultures were established as described before [62]. Cerebral cortices were removed from P1-3 C57BL/6 J mice. The cortices were digested with TrypLE™ Express Enzyme (Gibco) for 15 min at 37 °C. The dissociated cells were rinsed and resuspended in DMEM (Gibco) containing 10% fetal bovine serum and 1% antibiotic-antimycotic (Gibco). Subsequently, the viable cells (2 × 10^6^ cells) were plated on 75 cm^2^ flasks. Cultures were maintained in a 5% CO_2_ atmosphere at 37 °C and refed every 3 d throughout the study. Upon reaching confluency, astrocytes were seeded into 60 mm dishes at a density of 10^6^ cells per plate. And 10–14 day in cultures (DIV) were used for study.

Primary cortical neuronal cultures were established from embryonic days 14–16 mouse embryos (C57BL/6 J) as described previously [63]. Briefly, cortices were dissected in the ice-cold Hanks balanced salt solution. The tissue was treated with 0.25 mg/ml trypsin at 37 °C for 20 min. The cells were centrifuged at 1200 RPM at RT for 5 mins. The cell suspension was diluted in B-27 supplemented neurobasal medium containing 25 μM L-glutamine (Thermo Scientific) and 1% antibiotic-antimycotic (Gibco). The cells (1000 cells/mm^2^) were seeded on glass coverslips coated with poly-d-Lysine. Cultures were incubated at 37 °C in an incubator with 5% CO_2_ and atmospheric air. Cultures were fed every 3 days with fresh media and used for experiments at 7 DIV.

### In vitro ischemia of astrocyte cultures and drug treatment

In vitro ischemia was mimicked with well-established oxygen and glucose deprivation/reoxygenation (OGD/R) model as described previously [64]. Astrocyte cultures were rinsed twice with an isotonic OGD solution containing (in mM, pH 7.4): 0 glucose, 20 NaHCO_3_, 120 NaCl, 5.36 KCl, 0.33 Na_2_HPO_4_, 0.44 KH_2_PO_4_, 1.27 CaCl_2_, 0.81 MgSO_4_. Cells were incubated in OGD solution in a hypoxic incubator at 37^o^C (Forma model 3130, Thermo Forma, Marietta, OH) equilibrated with 95% N_2_ and 5% CO_2_ for 3 h. Normoxic control cells were incubated in 5% CO_2_ and atmospheric air in isotonic control buffer containing 5.5 mM glucose with the rest of the components in the buffer identical to the isotonic OGD solution. For reoxygenation (R), OGD-treated cells were incubated with DMEM and incubated at 37^o^C in 5% CO_2_ and atmospheric air.

In some experiments, astrocyte cultures were treated with ER stress inducer thapsigargin (100 nM) alone or in combination with GSK2606414 (5 μM), a potent inhibitor of PERK kinase signaling [18], or NHE1 inhibitor HOE642 (1 μM) for 24 h. To inhibit NOX activity, astrocytes were treated with general NOX inhibitor DPI or NOX4 specific inhibitor GKT137831 during OGD 3 h and Reox 1 h. We evaluated the dose-dependent effects of Re-LCN2 protein (1, 2, 4, and 6 µg/ml) on neuronal death and neurite degeneration of neuronal cultures. Re-LCN2 at a concentration of 2 µg/ml induced a significant increase in PI^+^ neurons but without increase in neurite degeneration (data not shown). A higher concentration of Re-LCN2 (4 µg/ml) significantly increased both neuronal death as well as neurite degeneration (Supplementary Fig. S6), which is consistent with previously published reports [16].

### Exosome isolation from ACM by Size Exclusion Chromatography (SEC)

Exosomes were isolated from astrocyte conditioned medium (ACM) obtained from astrocyte cultures at 10–14 DIV (4 × 10^6^cells/15 cm dish). ACM was centrifuged at 300 × *g* for 10 min at 4 °C to remove cell debris, followed by centrifugation at 2000 × *g* for 60 min at 4 °C and the supernatant was collected. The supernatant was further concentrated through Centricon Plus-70 Centrifugal Filter (Sigma, MO, USA) at 3500 × *g* for 30 min at 4 °C. The concentrate was filtered through a 0.22 μm filter (Celltreat Scientific Products, MA, USA) to remove contaminating apoptotic bodies and microvesicles, and was overlaid on qEV SEC columns (Izon Science, MA, USA) followed by elution with PBS. After discarding the void volume (~2.85 ml), six 500 µl fractions were collected following the manufacturer’s instruction and then each fraction was analyzed by nanoparticle tracking analysis (NTA) to pool EV-enriched fractions (f 1-3), while the pooled fractions 4–6 was non-EV fraction. The EV-enriched fraction (f 1-3) was further concentrated with Amicon Ultra-4 Centrifugal Filter Unit (Millipore, MA, USA) to obtain a final EV volume of ~60 µl.

### Isolation of exosomes from ischemic mouse brains

Exosomes were isolated from mouse brains as described previously with slight modifications [1]. Briefly, fresh frozen brains (CL and IL hemispheres) from wild-type and *Nhe1* Astro-KO mice at 24 h after ischemic stroke were sliced with a razor blade (on ice) to generate 2–3 mm^3^ sections. The sections were dissociated in the enzyme mixture from the neural dissociation kit (Miltenyi Biotec, Germany) and incubated at 37 °C for 20 min. After incubation, the samples were triturated slowly using a fire-polished pasteur pipette and the suspension was centrifuged at 300 g for 5 min at 4 °C. The pellet was collected (nominated as “total brain homogenate”) in lysis buffer (with protease and phosphatase inhibitors (PI/PS)), homogenized and stored at −80 °C for later use. The supernatant was transferred to a 15 ml-tube and then centrifuged at 2000 g for 10 min at 4 °C. This supernatant was then transferred to an eppendorf tube and then centrifuged at 10,000 g for 30 min at 4 °C. The supernatant was filtered through a 0.22 μm pore-size filter (Millipore) and overlaid on three sucrose cushions (2 ml each of 2.5 M, 1.3 M, and 0.6 M in 20 mM HEPES), then ultracentrifuged at 180,000 g for 3 h at 4 °C (Optima-XE SW41 Beckman Coulter) using SW41Ti rotor (Beckman). After the spin the top 1 ml of the gradient was discarded. Three 2 ml fractions were subsequently collected from the top to the bottom and the last 1 ml at the bottom of the tube was discarded. Each fraction was diluted with ice-cold PBS and spun at 100,000 × *g* at 4 °C (SW41Ti Beckman) for 1 h to pellet the vesicles. Following spin completion, the supernatant was discarded, and pellets were collected in ice-cold lysis buffer (with PI/PS). The vesicles in fractions 1–3 was characterized by NTA and TEM analysis to determine the nature of the vesicles and exosomes. The extracellular exosome vesicles ranging from 30 to 140 nm were identified in fraction 2 and equal quantities of exosome volume were used for immunoblotting.

### Nanoparticle tracking analysis (NTA)

Exosome concentration and size distribution were characterized by NTA with a NanoSight NS300 instrument (Malvern, Worcestershire, UK) and corresponding software version NTA3.4, Exosomes were diluted (1:100) in filtered sterile ddH2O to a final volume of 1 ml to achieve a concentration within 10^7^–10^8^ range for optimal analysis. Three videos of 30 s were captured under the following conditions: cell temperature: 22 °C; Syringe speed: 20 µl/s, camera level: 15. The mean size and exosome concentration (particles/ml) were calculated by integrating the data from three records adjusting a detecting threshold of 5.

### Transmission electron microscopy (TEM)

The isolated exosomes were centrifuged using an airfuge at 100,000 × *g* with 25 PSI for 45 min. The pellet was fixed with cold 2.5% v/v glutaraldehyde in 0.1 M PBS, rinsed in PBS, dehydrated through a graded series of ethanol and embedded in Epon. Ultra-thin sections (65 nm) were stained with uranyl acetate and Reynold’s lead citrate. A JEOL 1400 TEM was used for imaging at the Center for Biologic Imaging at the University of Pittsburgh.

### Neuronal viability assay

Neuronal viability in neuronal cultures was assessed by propidium iodide (PI) uptake and retention of calcein. Briefly, cultured neurons were rinsed with the HEPES-MEM buffer and incubated with 1 μg/ml calcein-AM and 10 μg/ml PI in the same buffer at 37 °C for 35 min. Cells were then rinsed with HEPES-MEM buffer and visualized using a Nikon TiE inverted epifluorescence microscope equipped with a 20× objective lens. Calcein or PI fluorescence was captured using ANDOR camera. Cells from each treatment group were counted and the viability was expressed as the ratio of calcein-positive cells to the sum of calcein-positive and PI-positive cells.

### Neuronal apoptosis and neurite degeneration

Neuronal cultures were fixed in 4% PFA and immunostaining was performed using rabbit anti-cleaved caspase-3 (1:100) and chicken anti-MAP-2 (1:2000) antibodies. Confocal images (3–5 images/ experimental condition) were acquired using NIKON A1R confocal microscope. The number of cells expressing the cleaved caspase-3 were counted and % of apoptosis was expressed as a ratio of caspase-3^+^/DAPI^+^ cells. The neurite degeneration was quantified by counting the number of MAP-2 expressing neurites showing focal swellings.

### Measurement of NADPH oxidase activity

NADPH oxidase activity assay was modified from Tarpey et al. [41]. Primary astrocytes treated with different conditions were washed twice with ice-cold PBS 1× solution (137 mM NaCl, 2.7 mM KCl, 10 mM Na_2_HPO_4_ and 1.8 mM KH_2_PO_4_), and then scraped from the petri dish and resuspended in 250 μl PBS. For the chemiluminescence assay, lucigenin (250 μM) and NADPH (150 μM) were added and incubated with 100 μl of samples with or without SOD (15 IU) and the luminescence was recorded using a luminometer every 15 mins (PerkinElmer, Victor Nivo^TM^). The NADPH induced the highest luminescence value minus the basal value was the NADPH oxidase activity. The value was expressed as Relative Light Units (RLU)/min.

### ROS measurement

ROS measurement was performed using the nonfluorescent cell-permeant compound 2’-7’-dichlorofluorescein diacetate (DCFH-DA) according to manufacturer’s instructions. DCFH-DA is hydrolyzed by intracellular esterases to dichlorofluorescin (DCFH) which is trapped within the cell. This nonfluorescent molecule is then oxidized to fluorescent dichlorofluorescin by action of cellular oxidants. Primary astrocytes treated with different conditions were washed twice with PBS 1× solution and cells were loaded with 5 μM DCFH-DA diluted in serum free media for 30 min at 37 °C. Cells were washed three times with PBS and fluorescent images were captured using a Nikon TiE inverted epifluorescence microscope equipped with a 20× objective lens.

### Histochemistry for detection of iron

For the detection of non-heme (mostly Fe^3+^ form) iron the DAB-enhanced Perl’s iron stain was used, as described previously with some modifications [65]. Briefly, brain sections were mounted on glass slides and allowed to dry overnight. The slides were washed in 0.01 M PBS and endogenous peroxidases were blocked by incubation with 0.3% H_2_O_2_ for 30 min. Subsequently, sections were washed again in PBS, and Perl’s solution (1:1 mix of 5% potassium ferrocyanide and 5% HCL both prepared in milliQ H_2_O) was prepared shortly before use. Sections were then incubated in Perl’s solution for 12 h and washed in milliQ H_2_O for 15 min. Diaminobenzidine (DAB) reaction was performed by incubating the sections with DAB/H_2_O_2_ solution (Vector labs) for 30 min followed by quick washing with PBS. The sections were dehydrated and coverslipped using DPX mounting media. Iron staining was performed in dark as much as possible.

### Statistical Analysis

A total of 60 mice (male and female, 3–4 months old) were used in the study. Sample sizes were determined based on pilot studies or our previously published work. All mice were randomly assigned to the procedures including both male and females in each group. All data analyses were performed by the investigators blinded to mouse genotypes and/or experimental group assignments. Mean values were reported together with the standard deviation (SD). Student’s two-tailed *t* test was used for comparison of two experimental groups. For three or more groups, multiple comparisons were done using one-way ANOVA where appropriate followed by appropriate comparisons test for multiple pairwise examinations. Changes were identified as statisticaly significant if p values were less than 0.05. Data was graphed using GraphPad Prism, version 8 (GraphPad Software, Inc., San Diego, CA) or Sigma plot (Systat Software Inc. San Jose, CA).

## Supplementary information

Supplementary information

Original Western Blotting Data File

Original Data File

Author agreement

Reproducibility checklist

## Data Availability

Data can be made available upon valid request.
