# Supplementary material for: NOX activation in reactive astrocytes regulates astrocytic LCN2 expression and neurodegeneration
Source: Cell Death Dis. 2022 Apr 19;13(4):371. doi: 10.1038/s41419-022-04831-8 (PMC9018876; doi:10.1038/s41419-022-04831-8)
Supplement: Supplementary file 1 — Supplementary information [file 41419_2022_4831_MOESM1_ESM.doc]

**NOX activation in reactive astrocytes regulates astrocytic LCN2 expression and neurodegeneration**

Ruijia Liu^1, 2^, Jun Wang^2^, Yang Chen^2,3^, Jenelle M. Collier^2,3^, Okan Capuk^2^, ^3^, Shijie Jin^4^, Ming Sun^6^, Sujan K. Mondal^5^, Theresa L. Whiteside^5^, Donna B. Stolz^6^, Yongjie Yang^4^, Gulnaz Begum^2,3*^

**Supplementary Information**

**Supplementary Table 1. List of antibodies and application**

| **Antibody** | **Species** | **Dilution** | **Company** | **Catalog No.** | **Application** |
| --- | --- | --- | --- | --- | --- |
| β-actin | Rb | 1:5000 | Cell Signaling Technology | 4970S | WB |
| GAPDH | Rb | 1:4000 | Cell Signaling Technology | 2118S |
| eIF2α | Ms | 1:1000 | Cell Signaling Technology | 2103S |
| CHOP | Ms | 1:1000 | Cell Signaling Technology | 2895S |
| ATF4 | Ms | 1:500 | Sigma life science | WH0000468M1 |
| Lipocalin-2 | Rb | 1:500 | Abcam | ab63929 |
| p-eIF2α  GADD34 | Rb  Rb | 1:1000  1:1000 | Abcam  Proteintech | ab32157  10449-1-AP |
| GFAP | Ms | 1:200 | Cell Signaling Technology | 3670S | IF |
| cleaved caspase 3 | Rb | 1:100 | Cell Signaling Technology | 9661S |
| NeuN | Ms | 1:200 | Millipore | MAB377 |
| Lipocalin-2 | Rat | 1:200 | Abcam | ab70287 |
| GRP78  NOX4 | Rb  Rb | 1:100  1:100 | Abcam  Abcam | ab21685  ab133303 |
| MAP2 | Chicken | 1:2000 | Novus Biologicals | NB300-213 |
| GFAP | Rb | 1:200 | Dako | z0334 |
| rmLCN2 | Ms | 4 μg/ml | R&D Systems, Inc. | 1857-LC-050 | Cell treatment |
| Lipocalin-2 | Ms | 10 μg/ml | R&D Systems, Inc. | MAB1857-100 |

**
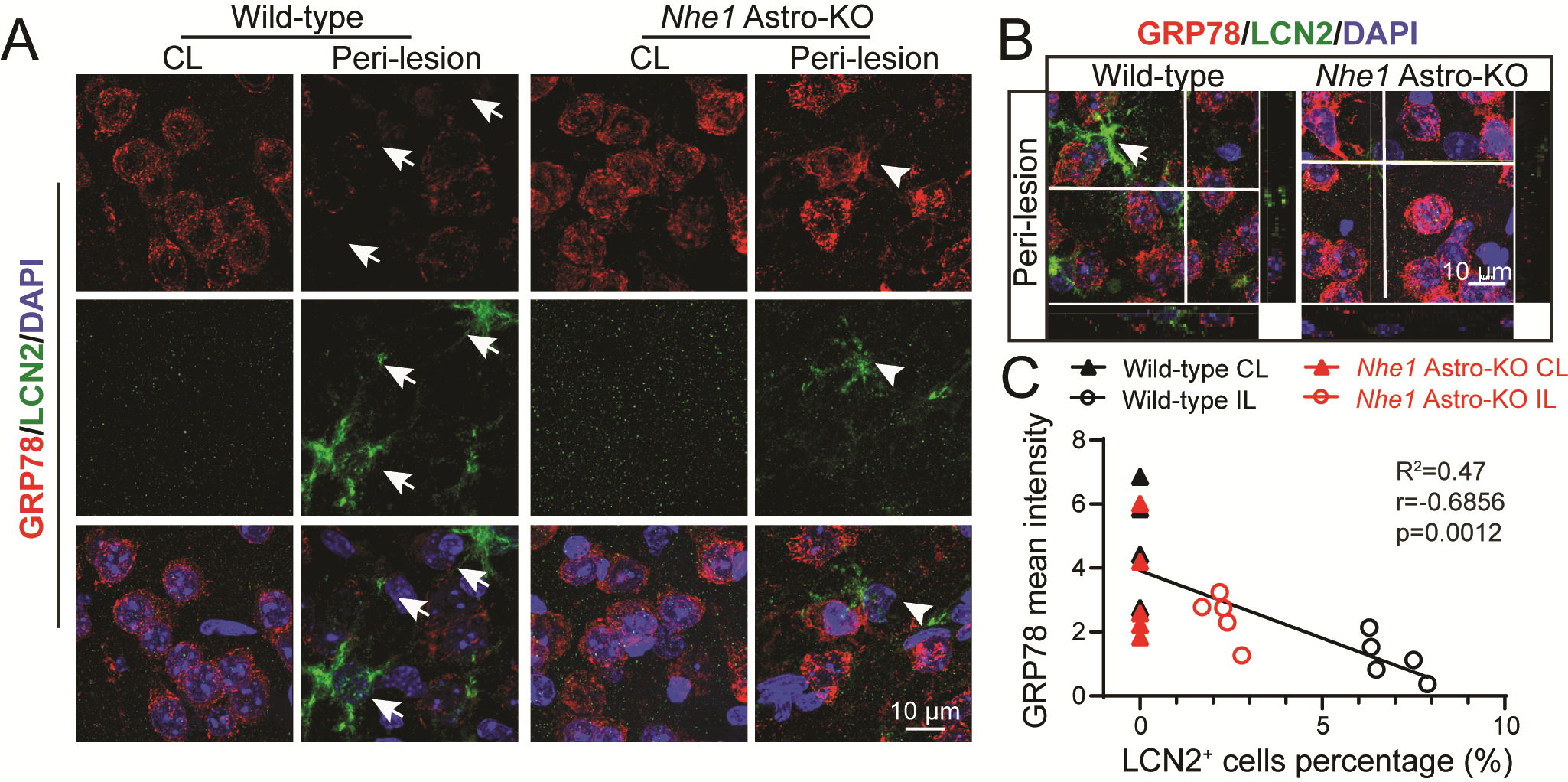
**

**Supplementary Figure 1. Increased LCN2 expression is associated with GRP78^+^ cell loss.**

**A.** Representative confocal images of GRP78 and LCN2 protein expression in WT and *Nhe1* Astro-KO brains at 48 h Rp. **Arrows**: high expression. **Arrowheads:** low expression. **(B)** Orthogonal sections from z stack confocal images showing association of NeuN^+^ neurons with LCN2^+^ astrocytes (arrowheads). **(C)** Negative correlation between LCN2 expression and GRP78^+^ intensity in wild-type and *Nhe1* Astro-KO brains. n=5; r =-0.6856, p=0.0012.

**
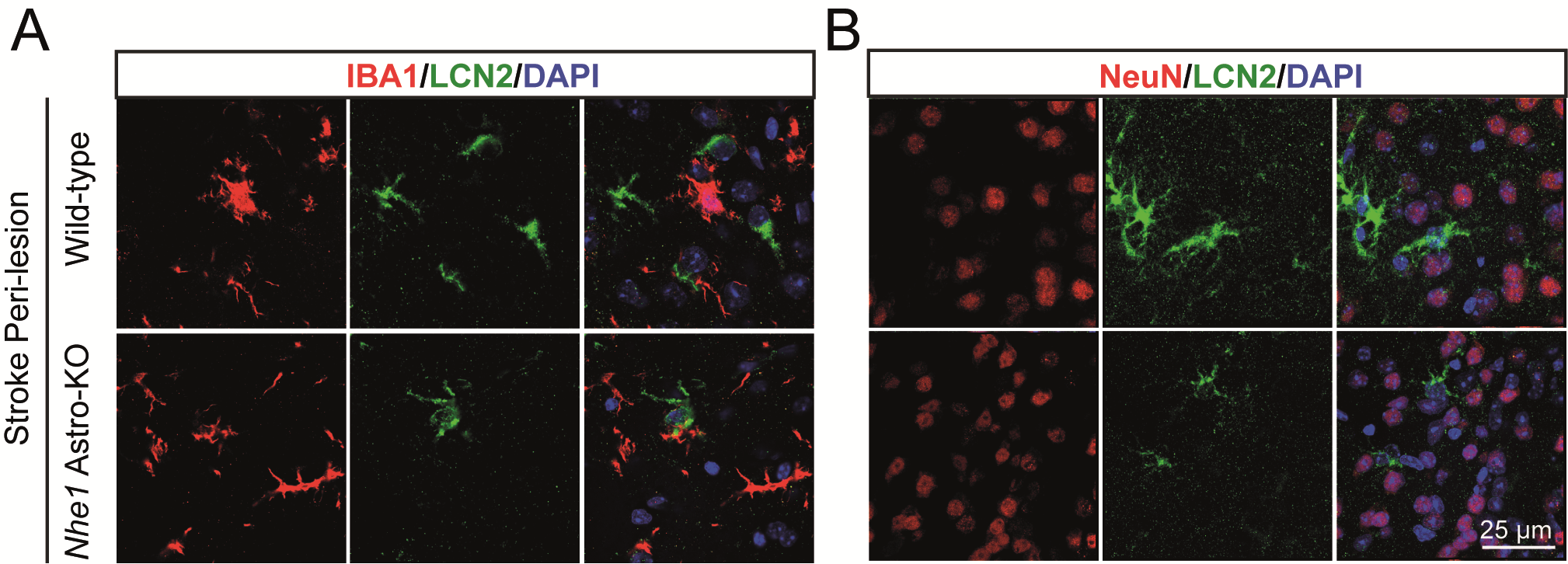
**

**Supplementary Figure 2. LCN2 is not expressed in neurons or microglial cells.**

**A.** Representative confocal images of IBA1 and LCN2 expression in microglia of wild-type and *Nhe1* Astro-KO brains at 48 h Rp. **B.** Representative confocal images of NeuN and LCN2 in peri-lesion areas of wild-type and *Nhe1* Astro-KO brains at 48 h Rp.


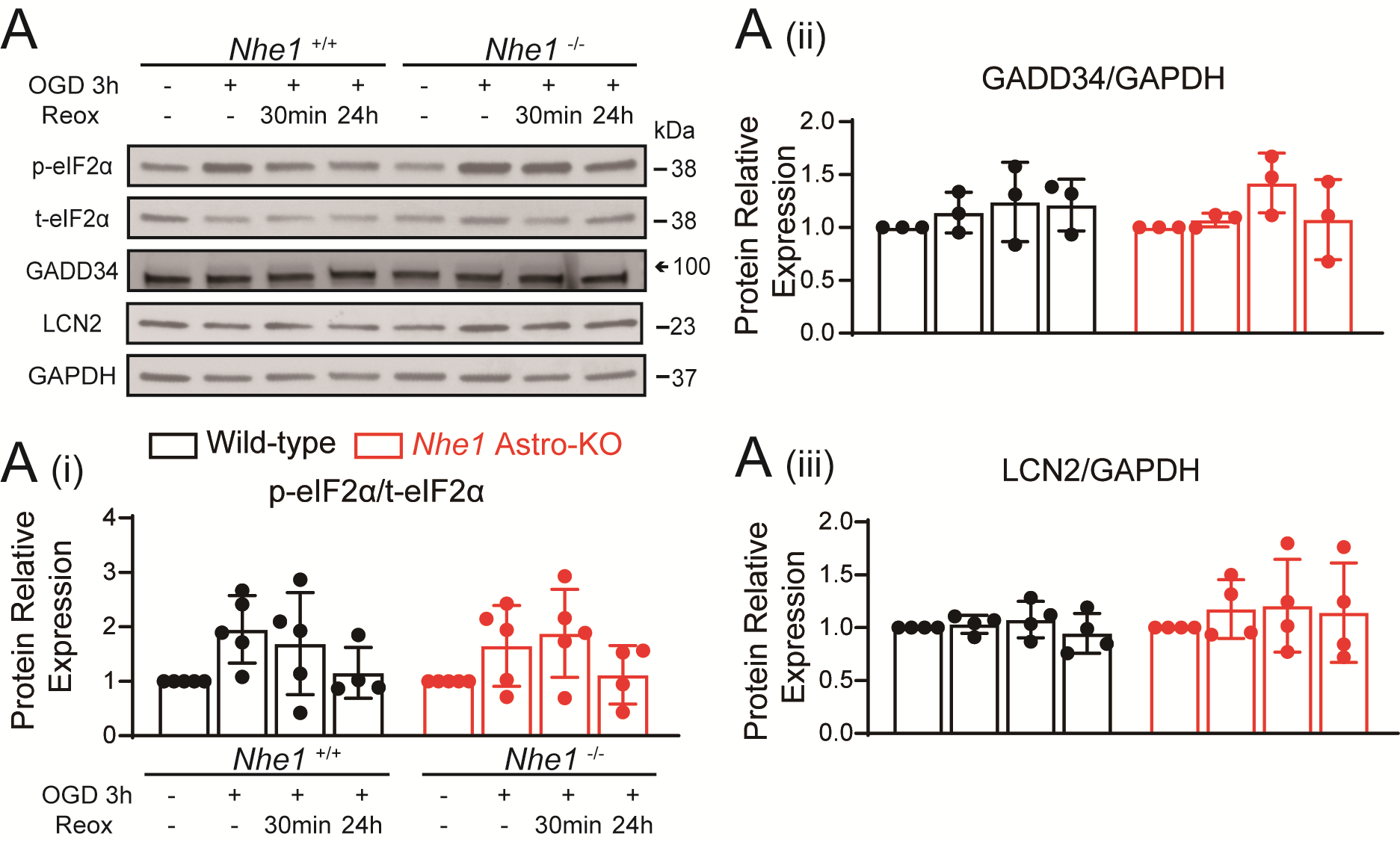


**Supplementary Figure 3. No changes in ER stress protein expression were detected in cultured *Nhe1^+/+^* or *Nhe1^-/-^* astrocytes subjected to OGD or OGD/R. (A)** Representative western blotting image showing the expression of ER stress protein and LCN2 in *Nhe1^+/+^* or *Nhe1^-/-^*astrocyte cultures subjected to normoxia, OGD or OGD/R. **A (i-iii)** Bar graph represents quantification of p-eIF2α, GADD34 and LCN2 band intensity. Data are mean ± SD, n=4.


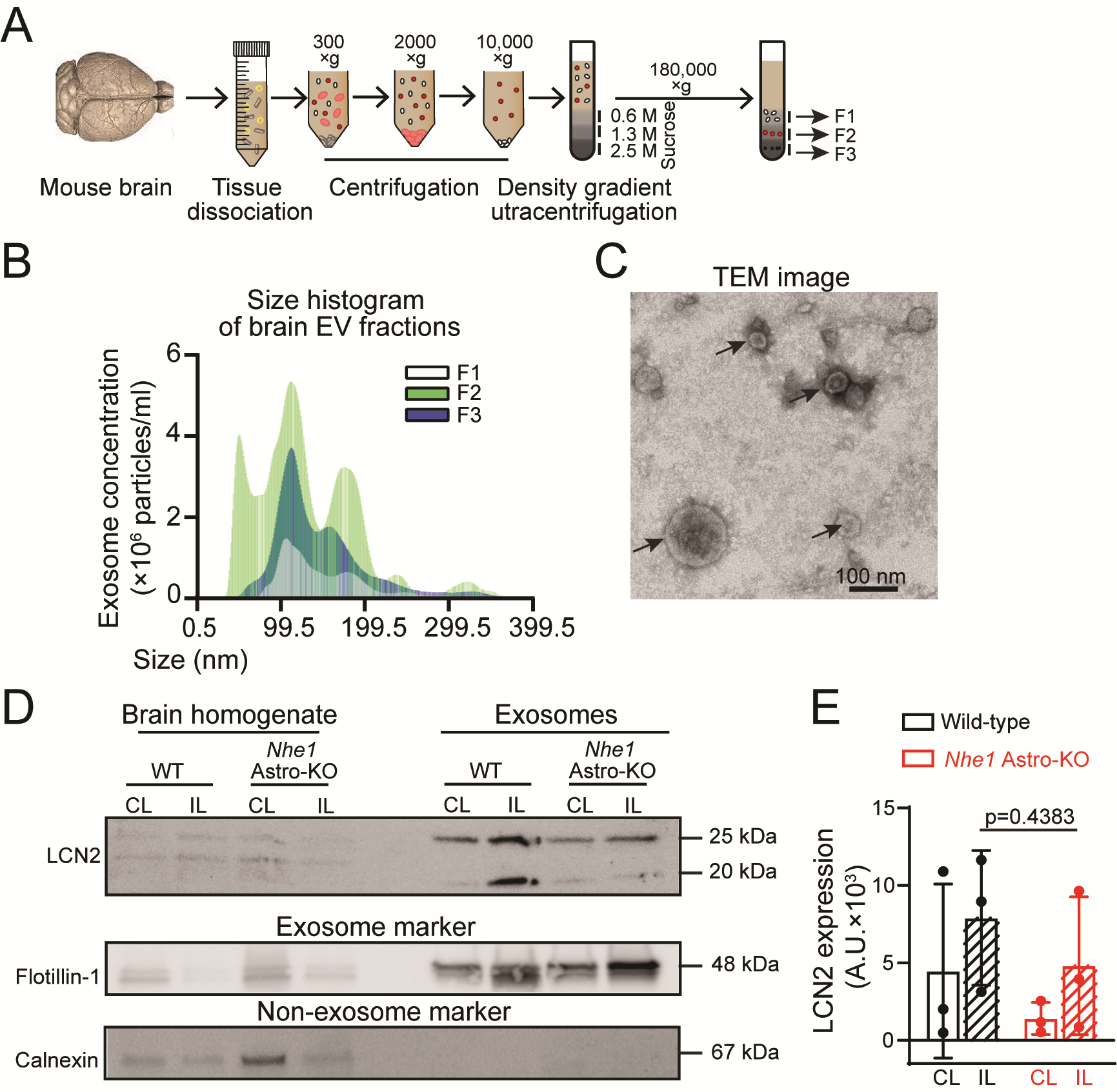


**Supplementary Figure 4. LCN2 expression in exosomes isolated from WT and *Nhe1* Astro-Ko mouse brains after ischemic stroke. (A)** Cartoon illustrates exosome isolation. **(B)** Representative size distribution and concentration analysis by NTA of extracellular vesicle (EV) fractions derived from sucrose gradient ultracentrifugation. Exosomes were enriched in fraction 2 (F2). **(C)** Representative TEM images of exosomes from F2. **(D)** Representative western blotting images of LCN2, exosome marker, or subcellular organelle marker expression in brain homogenate and exosome F2. Data are mean ± SD, n=3.

**
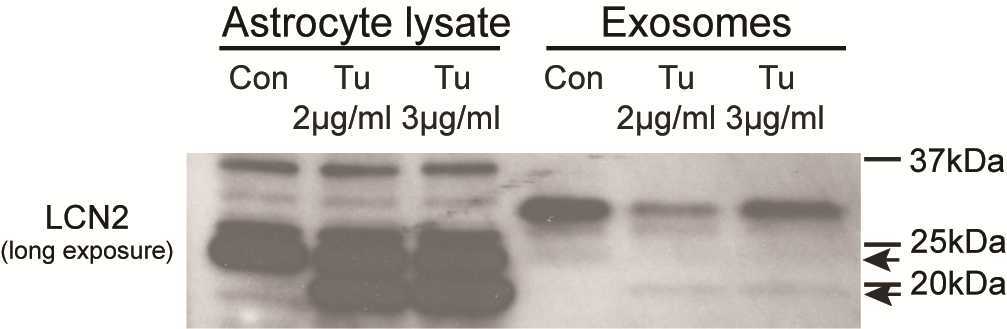
**

**Supplementary Figure 5. Tunicamycin-induced inhibition of glycosylation changed LCN2 protein band size.** Representative western blotting images showing increased expression of deglycosylated LCN2 proteins (20 and 25 kDa bands, **arrows**) in astrocyte lysates and exosomes treated with tunicamycin (2 or 3µg/ml).

**
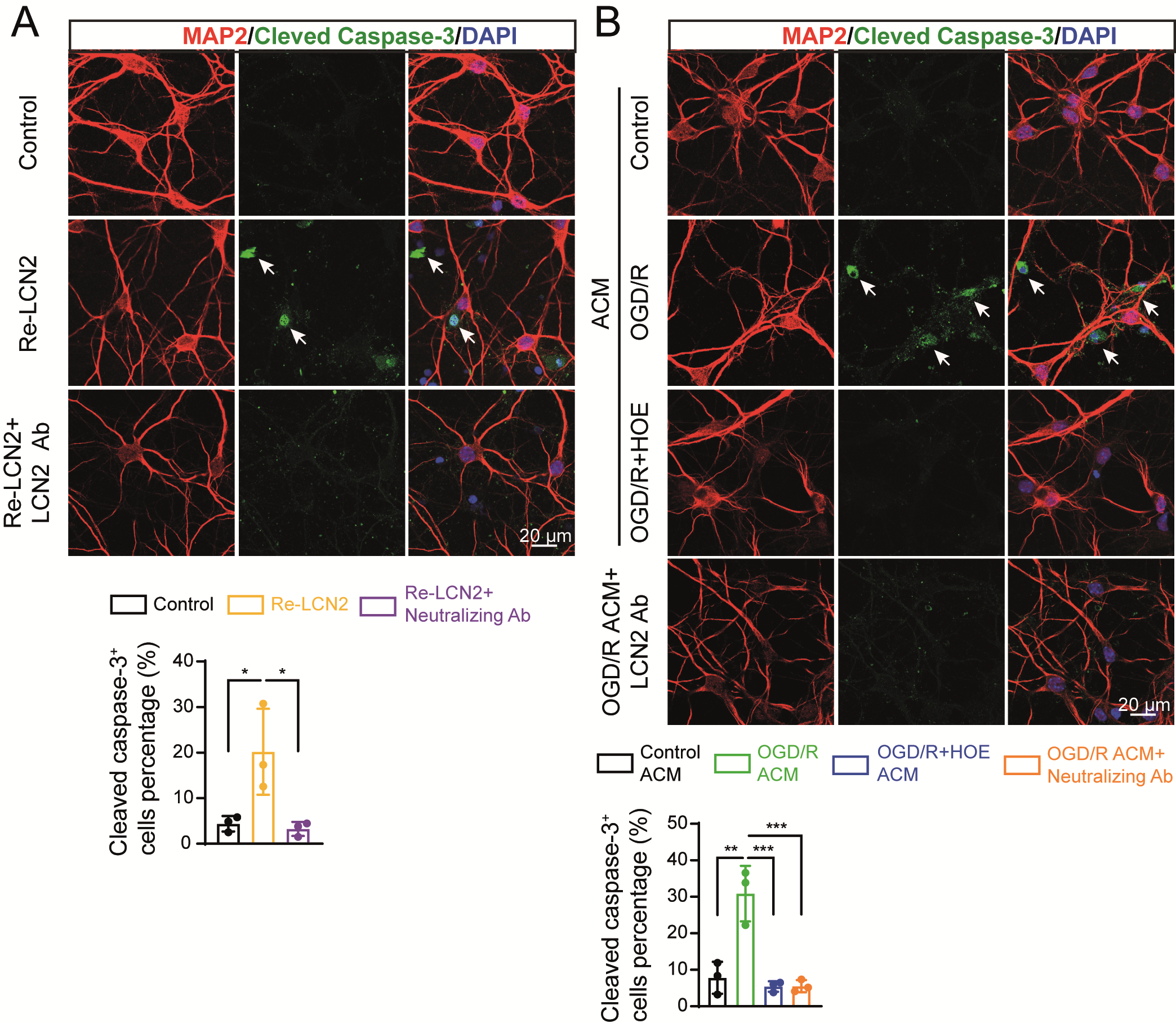
**

**Supplementary Figure 6. Cleaved caspase-3 in neurons was reduced upon neutralization of Re-LCN2 or OGD/R ACM with a monoclonal LCN2 antibody. (A)** Representative confocal images of MAP2 and cleaved caspase-3 stained neurons, treated with 4µg/ml Re-LCN2 or Re-LCN2^+^ neutralizing Ab for 48 h. **Arrows**: high expression. Sumamry data shows quantification of percentage of cleaved caspase-3 expression. Data are mean ± SD, n=3. *p < 0.05 via one-way ANOVA. **(B)** Representative MAP2^+^/cleaved caspase-3^+^ staining of primary neurons treated with ACM of control, OGD/R, OGD/R+HOE642, or OGD/R ACM + LCN2 neutralizing Ab groups. **Arrows**: high expression. Sumamry data shows quantification of percentage of cleaved caspase-3 expression. Data are mean ± SD, n=3. *p < 0.05; **p < 0.01; ***p < 0.001 via one-way ANOVA.

**
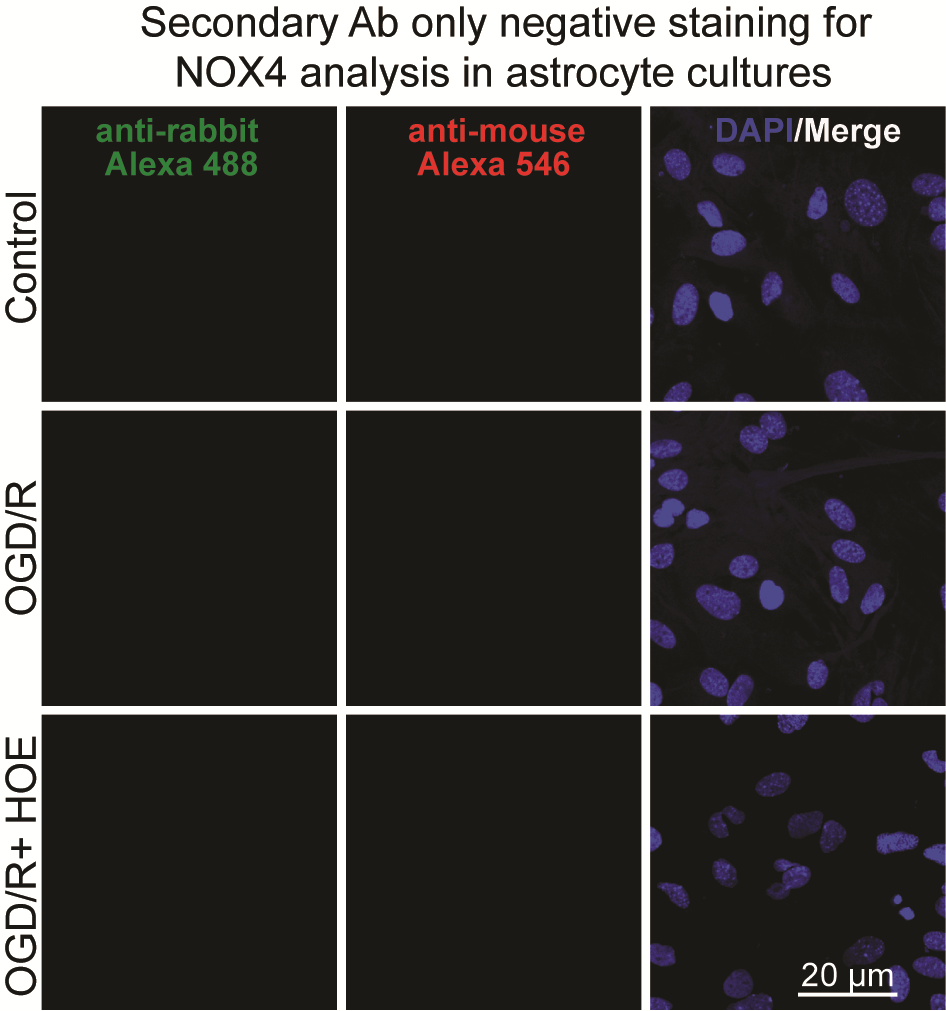
**

**Supplementary Figure 7. Representative negative control images.** Cultured astrocytes were stained with secondary antibodies only (Goat anti-rabbit Alexa 488 and Goat anti-mouse Alexa 546).
