# Supplementary material for: NOX activation in reactive astrocytes regulates astrocytic LCN2 expression and neurodegeneration
Source: Cell Death Dis. 2022 Apr 19;13(4):371. doi: 10.1038/s41419-022-04831-8 (PMC9018876; doi:10.1038/s41419-022-04831-8)

**ATF4 full-length blot of cultured astrocytes in Figure 3A**


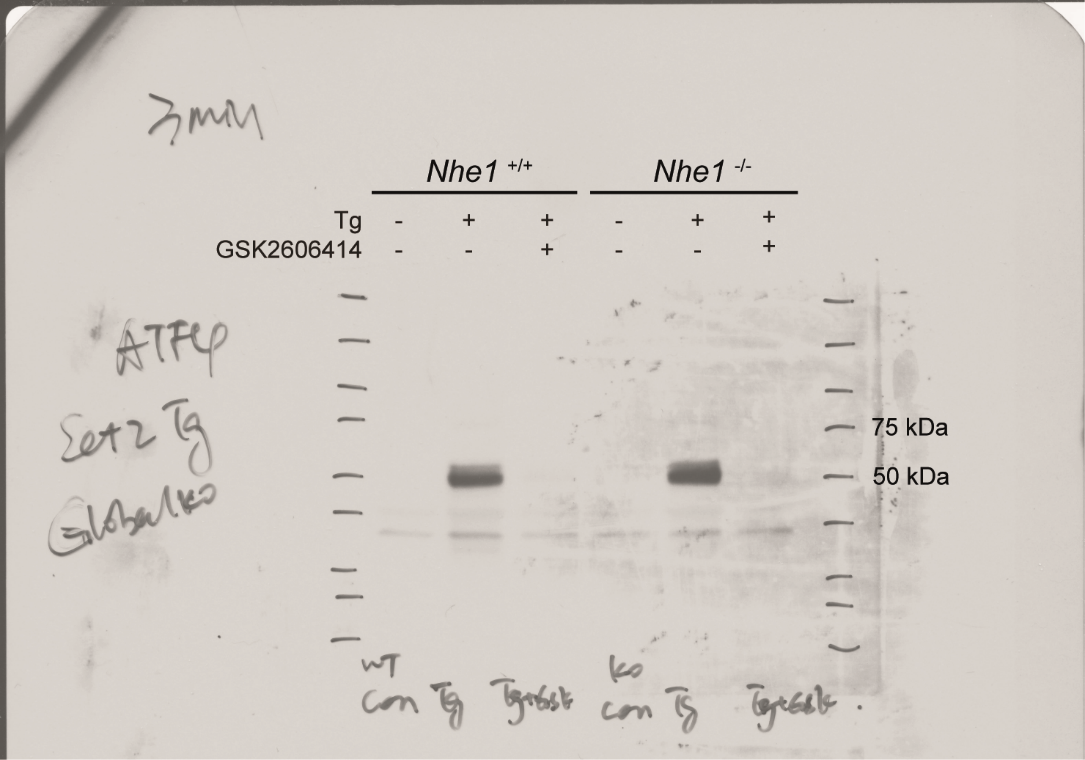


**GADD34 full-length blot of cultured astrocytes in Figure 3A**


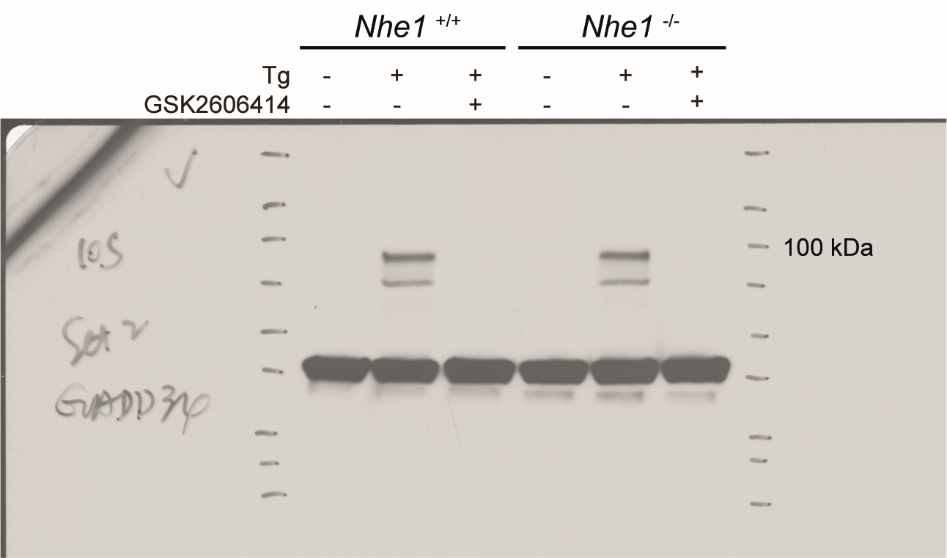


**LCN2 full-length blot of cultured astrocytes in Figure 3A**


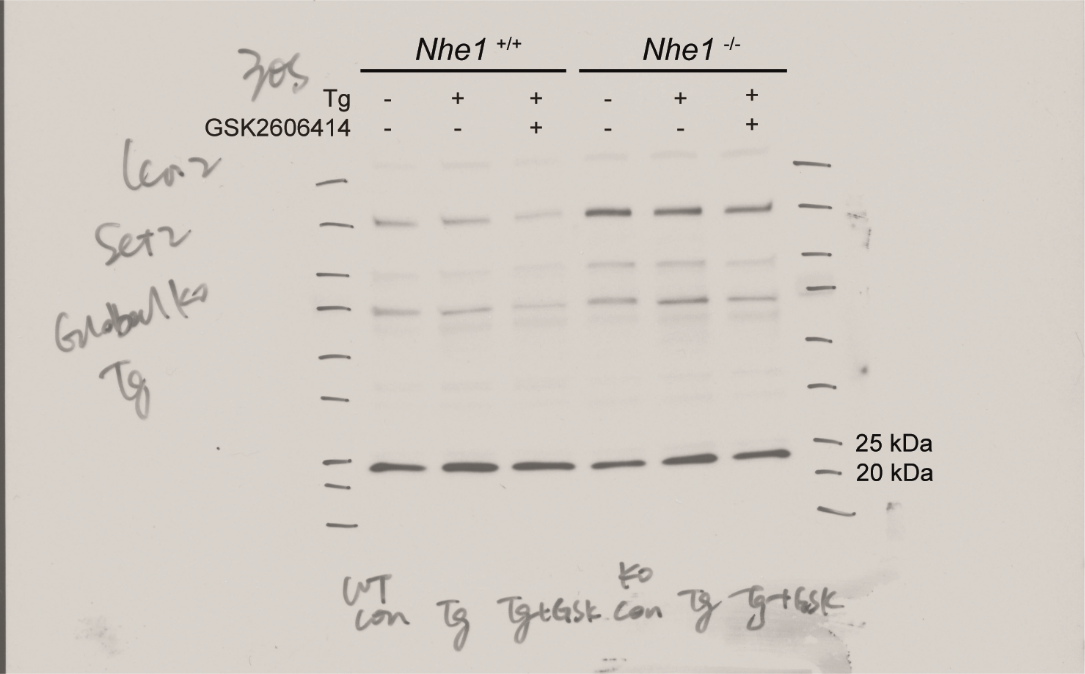


**GAPDH full-length blot of cultured astrocytes in Figure 3A**


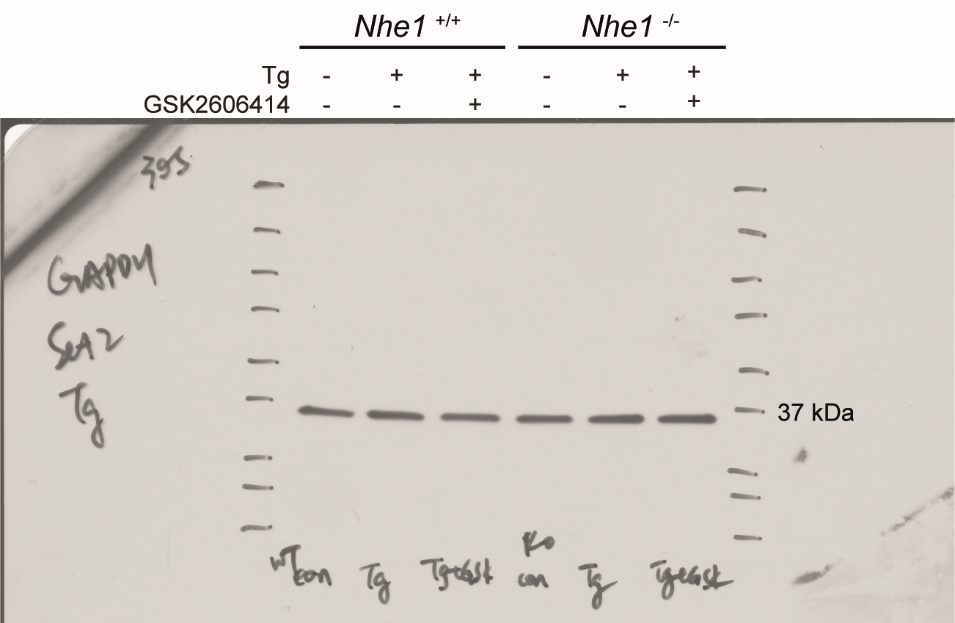


**p-eIF2α full-length blot of brain homogenates in Figure 3B**


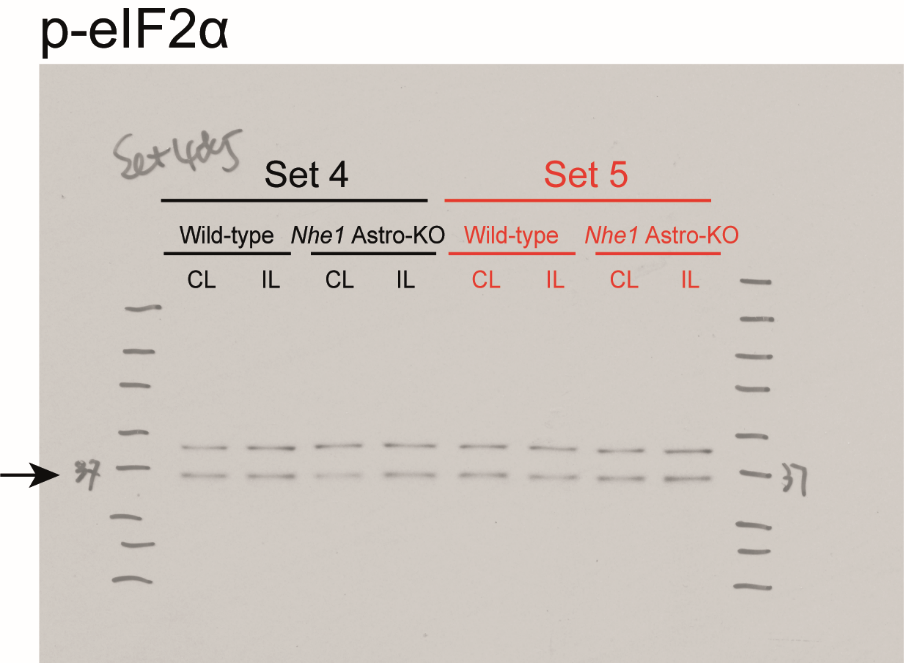


**t-eIF2α full-length blot of brain homogenates in Figure 3B**


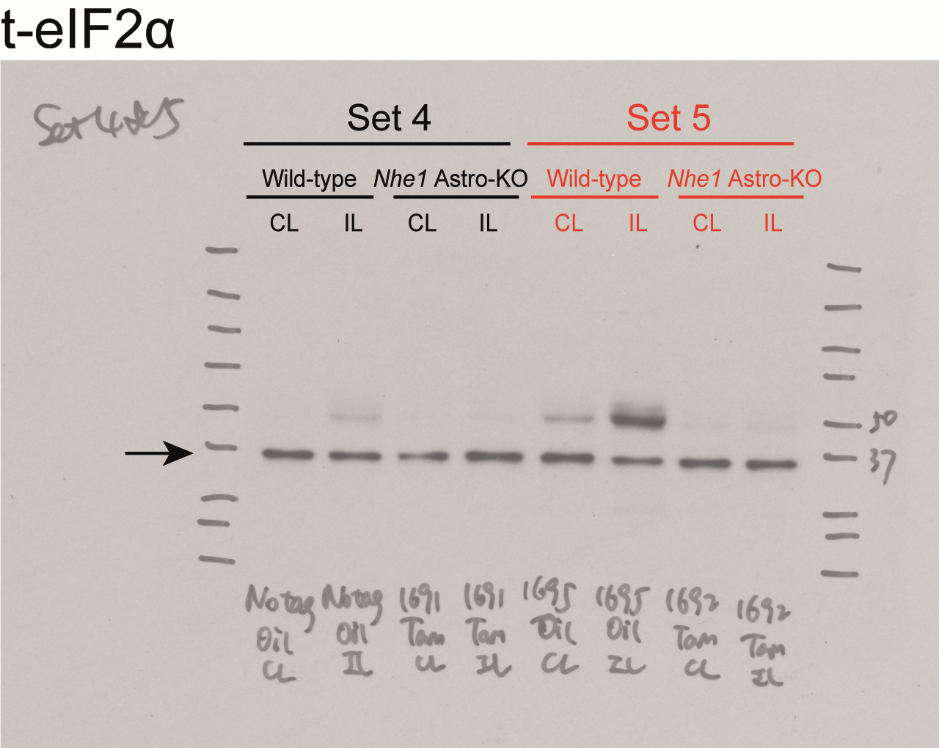


**ATF4 full-length blot of brain homogenates in Figure 3B**


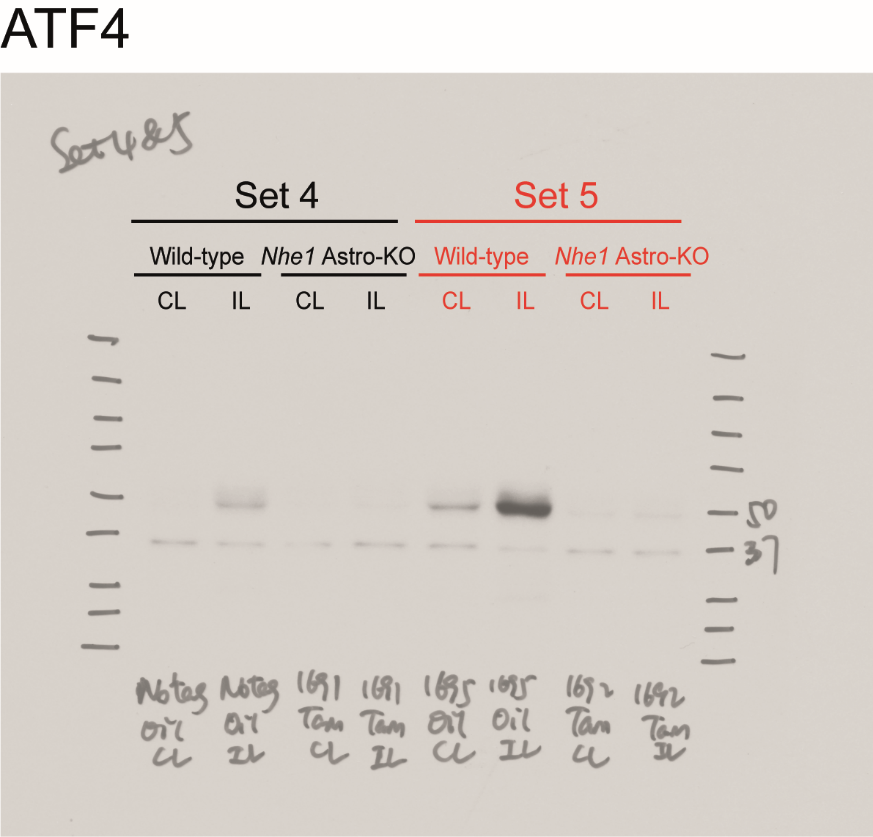


**CHOP full-length blot of brain homogenates in Figure 3B**


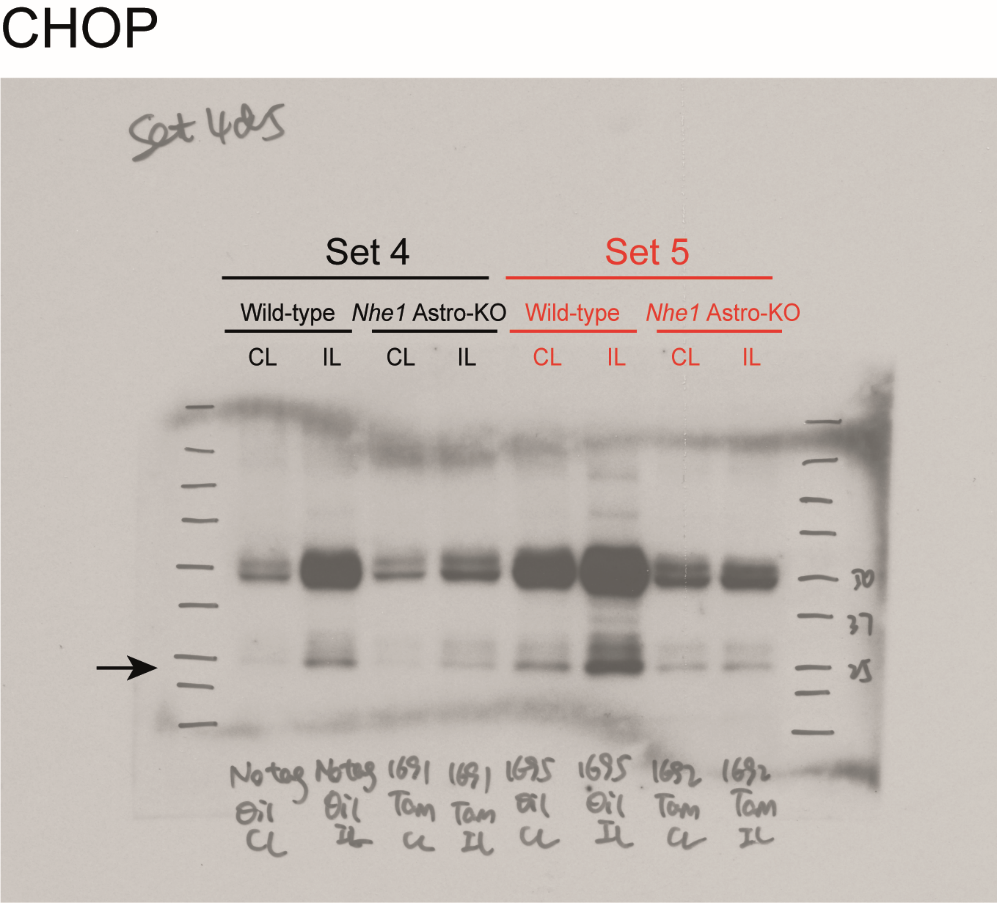


**β-actin full-length blot of brain homogenates in Figure 3B**


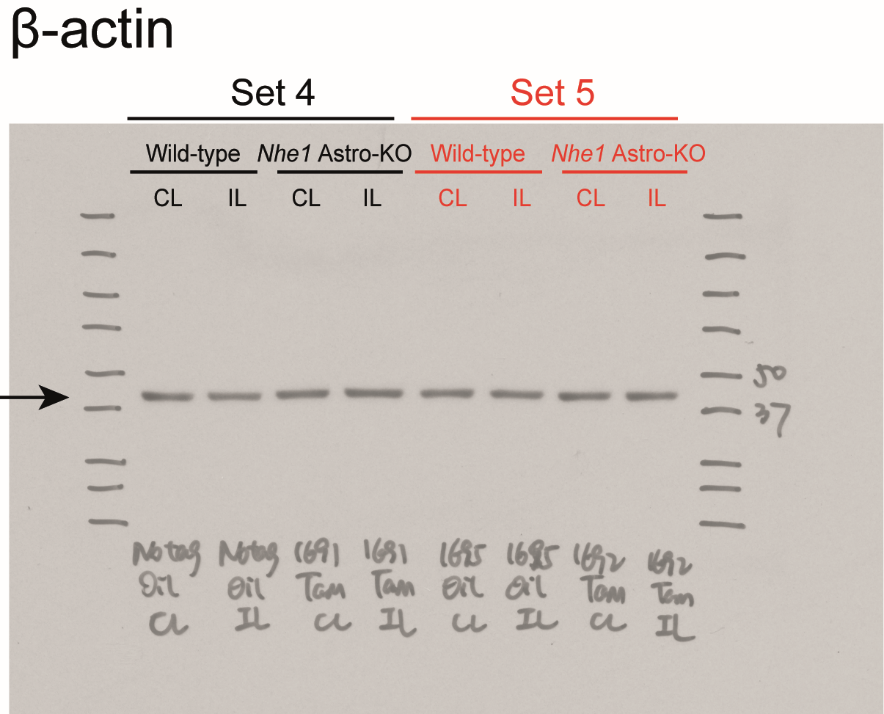


**p-eIF2α full-length blot of brain astrocytes in Figure 3C**


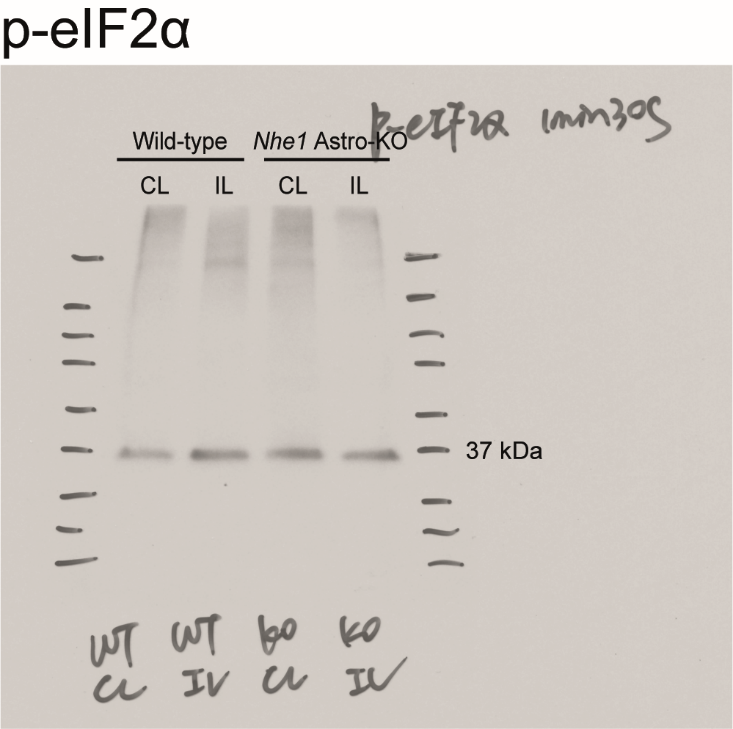


**t-eIF2α full-length blot of brain astrocytes in Figure 3C**


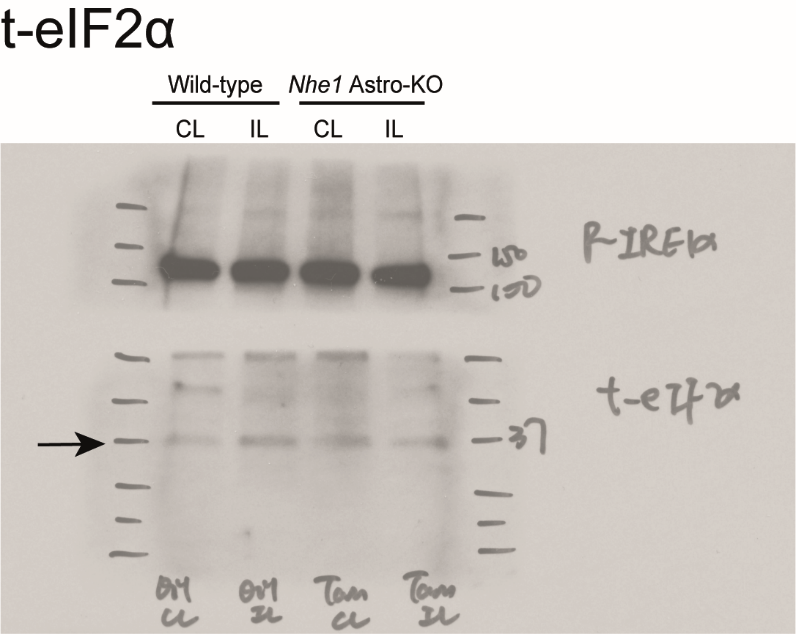


**ATF4 full-length blot of brain astrocytes in Figure 3C**


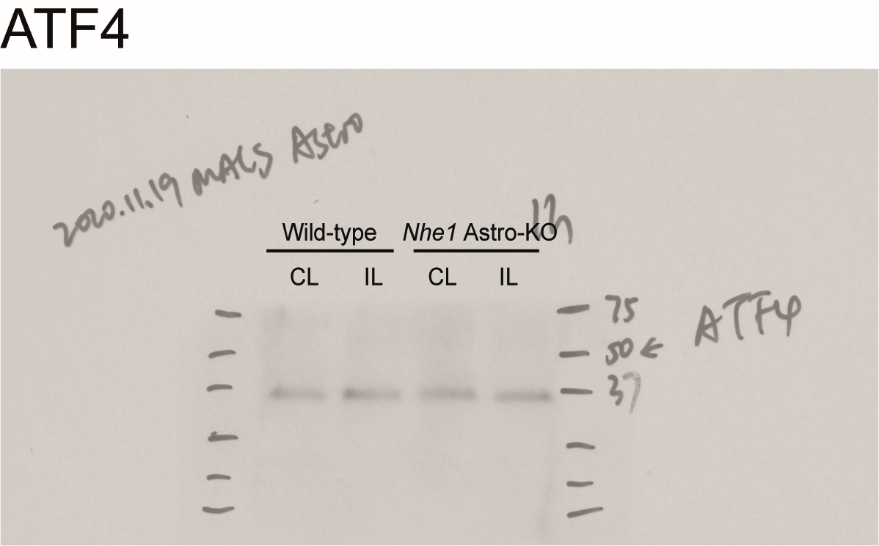


**CHOP full-length blot of brain astrocytes in Figure 3C**


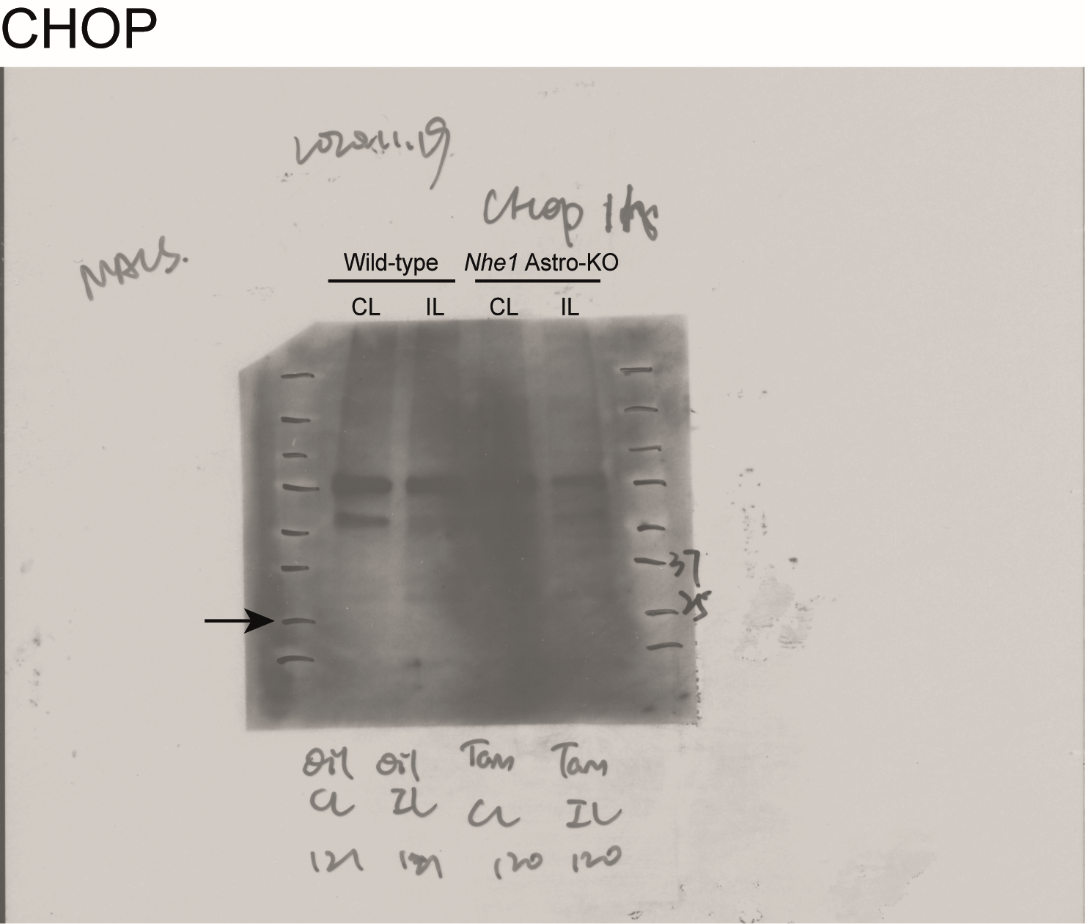


**GAPDH full-length blot of brain astrocytes in Figure 3C**


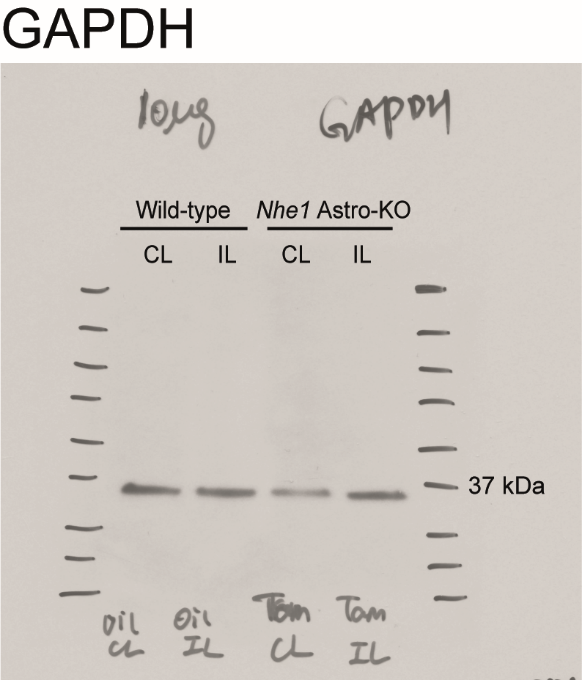


**CD81 full-length blot of exosomes in Figure 4C**


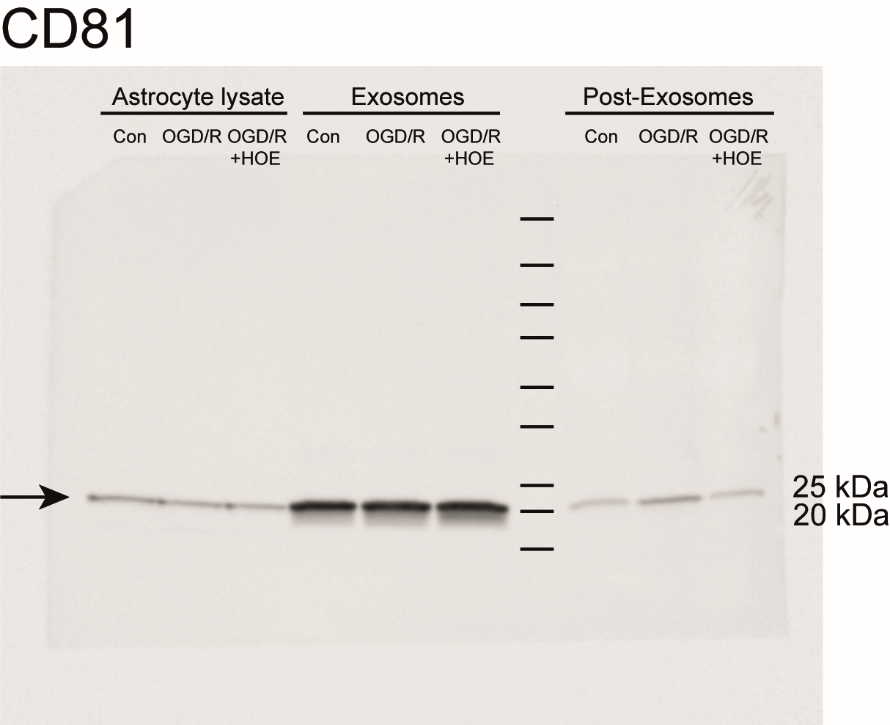


**Tsg101 full-length blot of exosomes in Figure 4C**


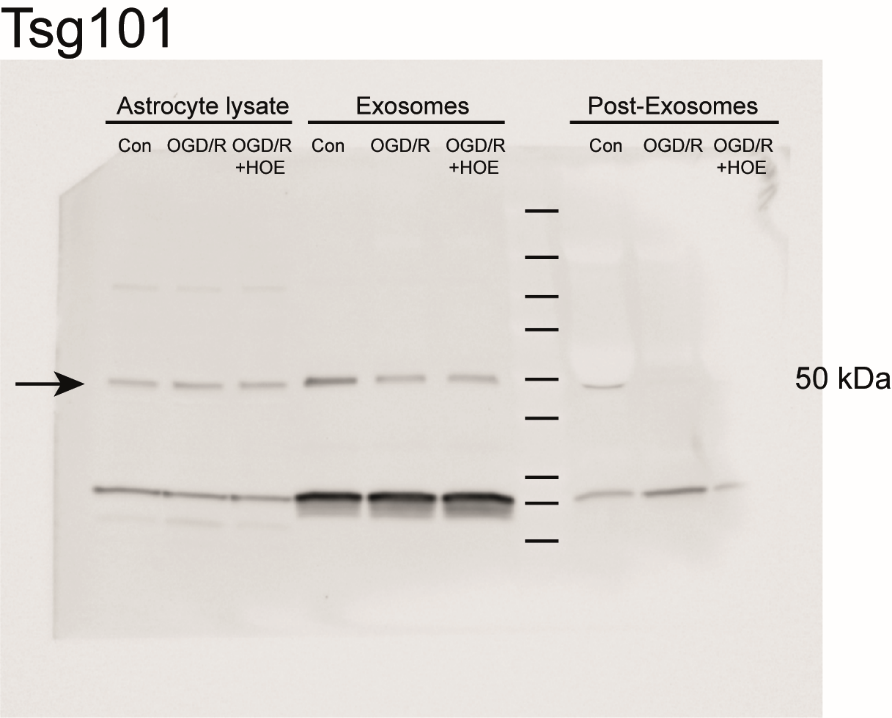


**Calregulin full-length blot of exosomes in Figure 4C**


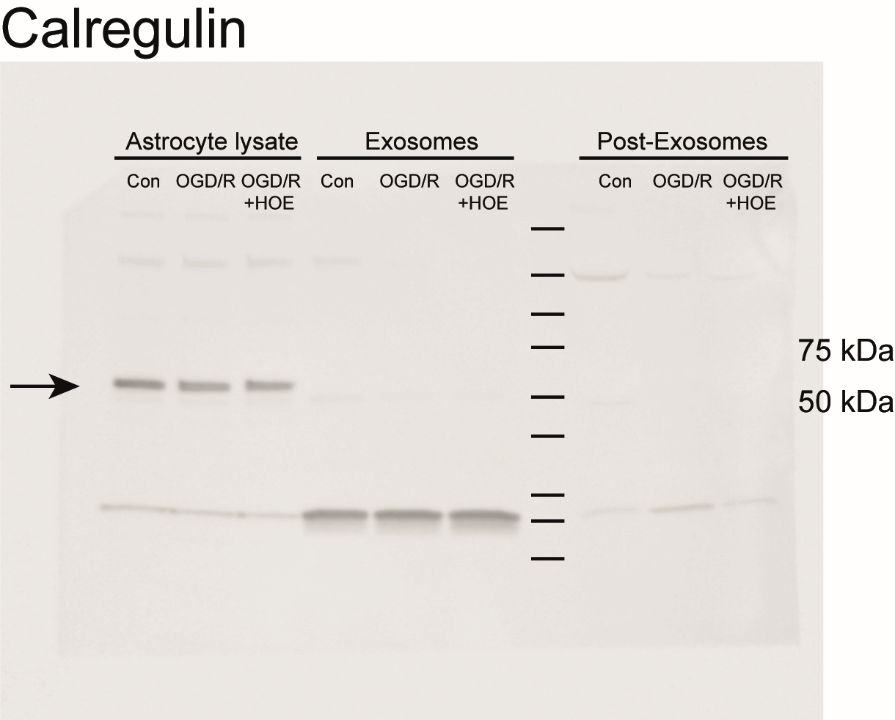


**EEA1 full-length blot of exosomes in Figure 4C**


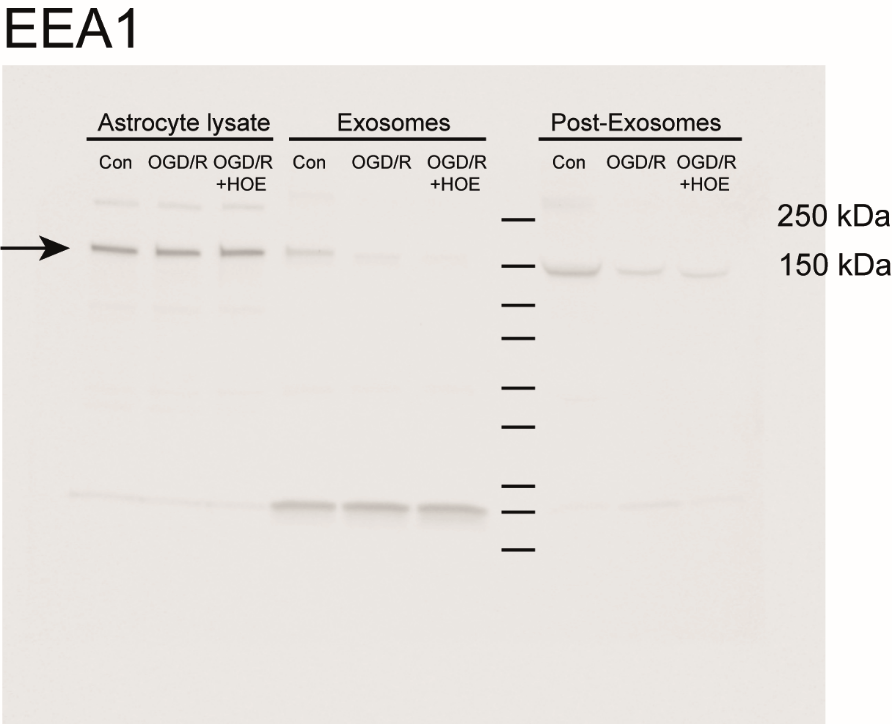


**Rab7 full-length blot of exosomes in Figure 4C**


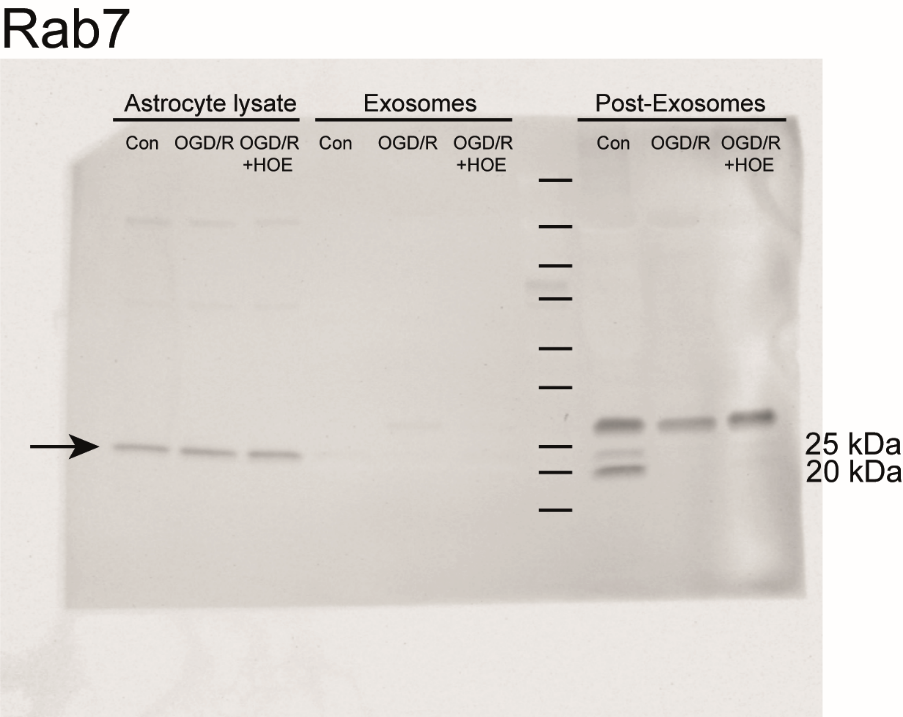


**GAPDH full-length blot of exosomes in Figure 4C**


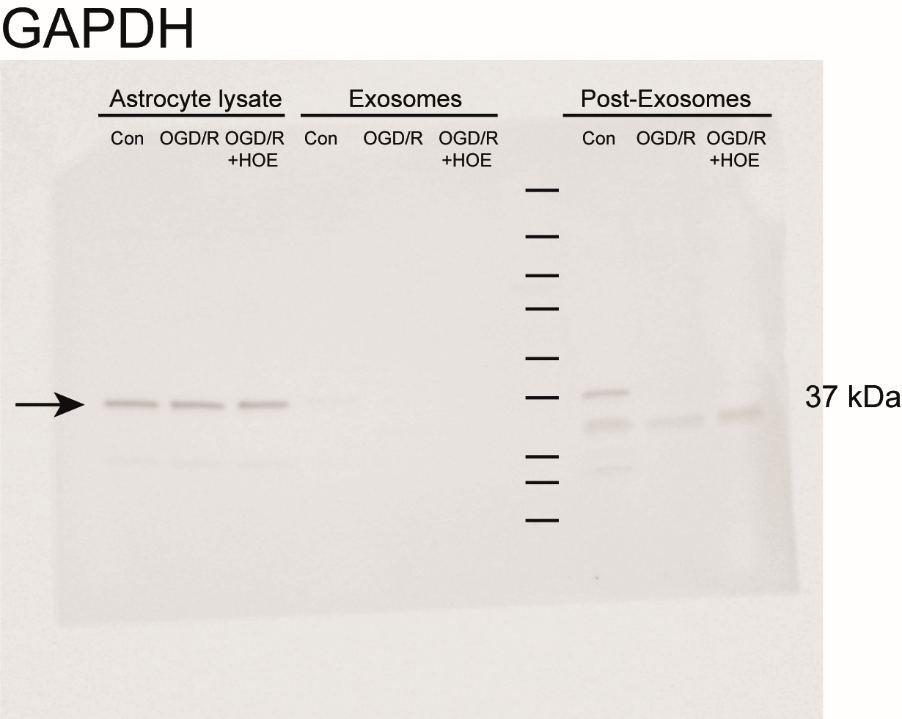


**LCN2 full-length blot of exosomes in Figure 4D**


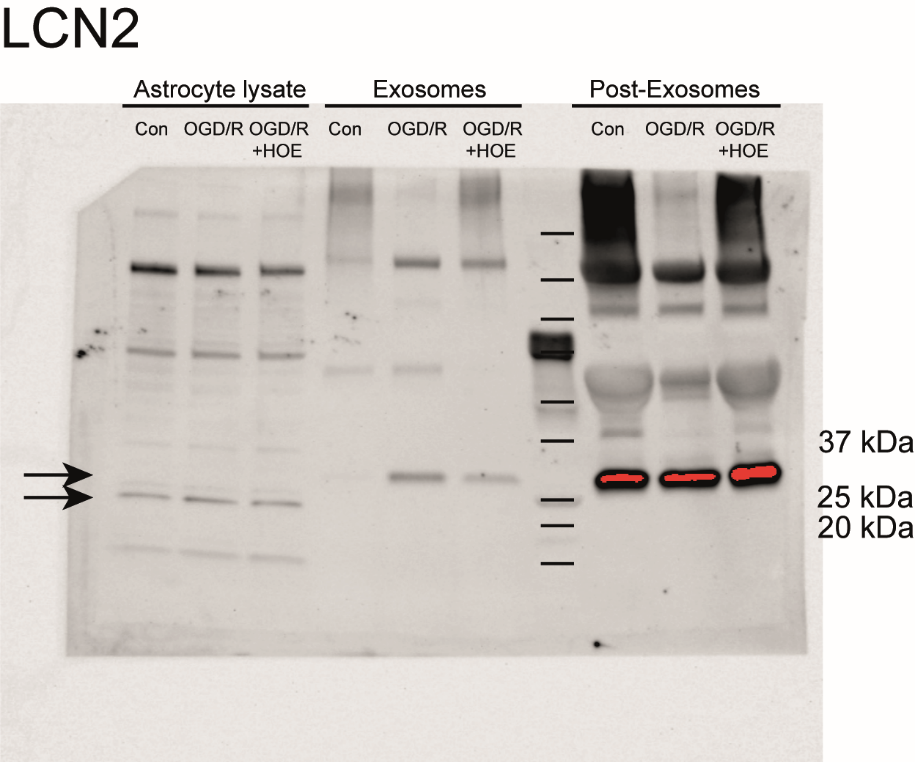


**LCN2 full-length blot of ACM in Figure 4E**


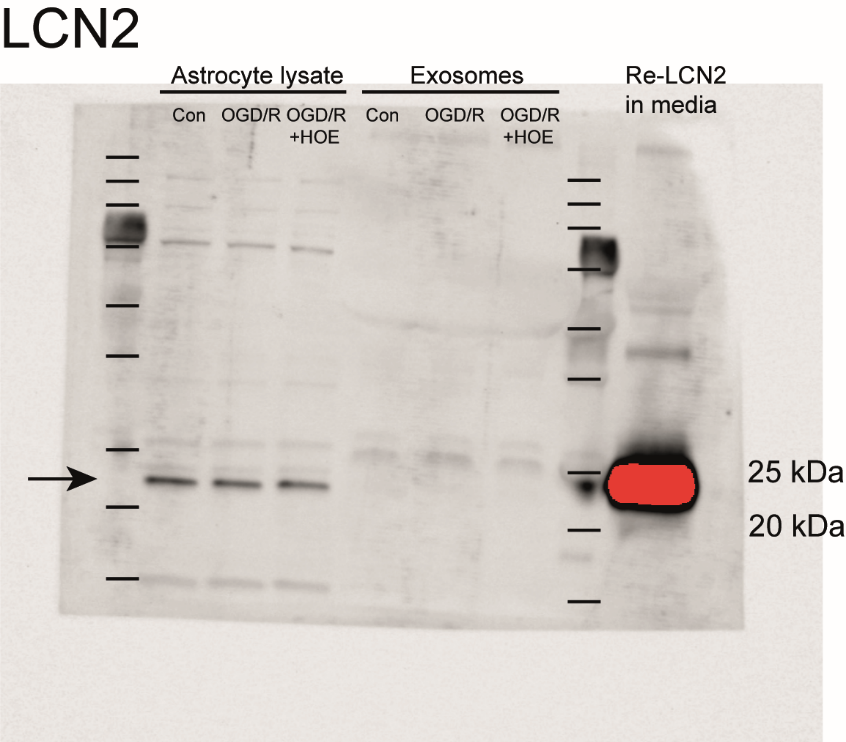

Supplement: Supplementary file 2 — Original Western Blotting Data File [file 41419_2022_4831_MOESM2_ESM.docx]
