# Supplementary material for: NOX activation in reactive astrocytes regulates astrocytic LCN2 expression and neurodegeneration
Source: Cell Death Dis. 2022 Apr 19;13(4):371. doi: 10.1038/s41419-022-04831-8 (PMC9018876; doi:10.1038/s41419-022-04831-8)
Supplement: Supplementary file 4 — Author agreement [file 41419_2022_4831_MOESM4_ESM.pdf]

Re: Author agreement_CDDIS-21-4158RR

Ruijia Liu <ruijiarachel@gmail.com>
Fri 4/1/2022 10:07 AM
To: Begum, Gulnaz <gub6@pitt.edu>

Hi Gulnaz,

I agree to the authorship inclusions/changes.

Thanks,
Ruijia

On Fri, Apr 1, 2022 at 9:50 PM Begum, Gulnaz <gub6@pitt.edu> wrote:

> Dear Ruijia,
>
> Our manuscript entitled "NOX activation in reactive astrocytes regulates astrocytic LCN2 expression and neurodegeneration" has been accepted for publication in Cell death and disease journal. There are following changes in the authorship: "Yang Chen"; "Okan Capuk"; "Ming Sun", and "Jenelle M Collier" have been included because they collected new experimental results in the re submission. Dandan Sun has been removed from the authorship based on her request. Please reply to this email stating you agree to the authorship inclusions/changes. Following is the order of the authors/co-authors as appear in the manuscript.
>
> "NOX activation in reactive astrocytes regulates astrocytic LCN2 expression and neurodegeneration"
>
> Ruijia Liu1, 2, Jun Wang2, Yang Chen2, Jenelle M. Collier2, 3, Okan Capuk2, Shijie Jin4, Ming Sun5, Sujan K. Mondal6, Theresa L. Whiteside6, Donna B. Stolz5, Yongjie Yang4, Gulnaz Begum2*
>
> Please reply to this email "**ASAP**"
>
> Thank you,
> Gulnaz Begum

Re: Author agreement_CDDIS-21-4158RR

Jun Wang <wangjdeh@163.com>
Fri 4/1/2022 8:32 PM
To: Begum, Gulnaz <gub6@pitt.edu>

Yes, I agree.

Thanks,

Jun

Jun Wang

邮箱：wangjdeh@163.com

签名由 网易邮箱大师 定制

On 04/02/2022 08:10, Begum, Gulnaz wrote:

Dear Jun,

Our manuscript entitled "NOX activation in reactive astrocytes regulates astrocytic LCN2 expression and neurodegeneration" has been accepted for publication in Cell death and disease journal. There are following changes in the authorship: "Yang Chen"; "Okan Capuk"; "Ming Sun", and "Jenelle M Collier" have been included because they collected new experimental results in the re submission. Dandan Sun has been removed from the authorship based on her request. Please reply to this email stating you agree to the authorship inclusions/changes. Following is the order of the authors/co-authors as appear in the manuscript.

"NOX activation in reactive astrocytes regulates astrocytic LCN2 expression and neurodegeneration"

Ruijia Liu1, 2, Jun Wang2, Yang Chen2, Jenelle M. Collier2, 3, Okan Capuk2, Shijie Jin4, Ming Sun5, Sujan K. Mondal6, Theresa L. Whiteside6, Donna B. Stolz5, Yongjie Yang4, Gulnaz Begum2*

Please reply to this email "**ASAP**"

Thank you,
Gulnaz Begum

Re: Author agreement_CDDIS-21-4158RR

Chen, Yang <YC21@pitt.edu>
Fri 4/1/2022 9:59 AM
To: Begum, Gulnaz <gub6@pitt.edu>

I agree. Thank you.
Yang

Get Outlook for Android

From: Begum, Gulnaz <gub6@pitt.edu>
Sent: Friday, April 1, 2022 9:52:27 AM
To: Chen, Yang <YC21@pitt.edu>
Subject: Author agreement_CDDIS-21-4158RR

Dear Yang,

Our manuscript entitled "NOX activation in reactive astrocytes regulates astrocytic LCN2 expression and neurodegeneration" has been accepted for publication in Cell death and disease journal. There are following changes in the authorship: "Yang Chen"; "Okan Capuk"; "Ming Sun", and "Jenelle M Collier" have been included because they collected new experimental results in the re submission. Dandan Sun has been removed from the authorship based on her request. Please reply to this email stating you agree to the authorship inclusions/changes. Following is the order of the authors/co-authors as appear in the manuscript.

"NOX activation in reactive astrocytes regulates astrocytic LCN2 expression and neurodegeneration"

Ruijia Liu1, 2, Jun Wang2, Yang Chen2, Jenelle M. Collier2, 3, Okan Capuk2, Shijie Jin4, Ming Sun5, Sujan K. Mondal6, Theresa L. Whiteside6, Donna B. Stolz5, Yongjie Yang4, Gulnaz Begum2*

Please reply to this email "**ASAP**"

Thank you,
Gulnaz Begum

Re: Author agreement_CDDIS-21-4158RR

Collier, Jenelle Monet <JMC357@pitt.edu>
Fri 4/1/2022 10:38 AM
To: Begum, Gulnaz <gub6@pitt.edu>

I agree with authorship changed

Jenelle M. Collier
Pre-Doctoral Fellow, CNUP
University of Pittsburgh

From: Begum, Gulnaz <gub6@pitt.edu>
Sent: Friday, April 1, 2022 9:51:18 AM
To: Collier, Jenelle Monet <JMC357@pitt.edu>
Subject: Author agreement_CDDIS-21-4158RR

Dear Jenelle,

Our manuscript entitled "NOX activation in reactive astrocytes regulates astrocytic LCN2 expression and neurodegeneration" has been accepted for publication in Cell death and disease journal. There are following changes in the authorship: "Yang Chen"; "Okan Capuk"; "Ming Sun", and "Jenelle M Collier" have been included because they collected new experimental results in the re submission. Dandan Sun has been removed from the authorship based on her request. Please reply to this email stating you agree to the authorship inclusions/changes. Following is the order of the authors/co-authors as appear in the manuscript.

"NOX activation in reactive astrocytes regulates astrocytic LCN2 expression and neurodegeneration"

Ruijia Liu1, 2, Jun Wang2, Yang Chen2, Jenelle M. Collier2, 3, Okan Capuk2, Shijie Jin4, Ming Sun5, Sujan K. Mondal6, Theresa L. Whiteside6, Donna B. Stolz5, Yongjie Yang4, Gulnaz Begum2*

Please reply to this email "**ASAP**"

Thank you,
Gulnaz Begum

Re: Author agreement_CDDIS-21-4158RR

Capuk, Okan <CAPUK@pitt.edu>
Fri 4/1/2022 11:07 AM
To: Begum, Gulnaz <gub6@pitt.edu>

Hi Gulnaz,

I agree to the authorship inclusions/changes.

Thank you.
Best,
Okan Capuk

Android için Outlook'u edinin

From: Begum, Gulnaz <gub6@pitt.edu>
Sent: Friday, April 1, 2022 9:51:55 AM
To: Capuk, Okan <CAPUK@pitt.edu>
Subject: Author agreement_CDDIS-21-4158RR

Dear Okan,

Our manuscript entitled "NOX activation in reactive astrocytes regulates astrocytic LCN2 expression and neurodegeneration" has been accepted for publication in Cell death and disease journal. There are following changes in the authorship: "Yang Chen"; "Okan Capuk"; "Ming Sun", and "Jenelle M Collier" have been included because they collected new experimental results in the re submission. Dandan Sun has been removed from the authorship based on her request. Please reply to this email stating you agree to the authorship inclusions/changes. Following is the order of the authors/co-authors as appear in the manuscript.

"NOX activation in reactive astrocytes regulates astrocytic LCN2 expression and neurodegeneration"

Ruijia Liu1, 2, Jun Wang2, Yang Chen2, Jenelle M. Collier2, 3, Okan Capuk2, Shijie Jin4, Ming Sun5, Sujan K. Mondal6, Theresa L. Whiteside6, Donna B. Stolz5, Yongjie Yang4, Gulnaz Begum2*

Please reply to this email "**ASAP**"

Thank you,
Gulnaz Begum

Re: Author agreement_CDDIS-21-4158RR

Shijie Jin <jin.shijie1977@gmail.com>
Fri 4/1/2022 12:17 PM
To: Begum, Gulnaz <gub6@pitt.edu>

Hi Gulnaz:

I agree with this authorship's inclusions/changes in the manuscript entitled "NOX activation in reactive astrocytes regulates astrocytic LCN2 expression and neurodegeneration"

Best regards,
Shijie

On Fri, Apr 1, 2022 at 9:40 AM Begum, Gulnaz <gub6@pitt.edu> wrote:

> Dear Shijie,
>
> Our manuscript entitled "NOX activation in reactive astrocytes regulates astrocytic LCN2 expression and neurodegeneration" has been accepted for publication in Cell death and disease journal. There are following changes in the authorship: Yang Chen; Okan Capuk; Ming Sun, and Jenelle M Collier have been included because they collected new experimental results in the re submission. Dandan Sun has been removed from the authorship based on her request. Please reply to this email stating you agree to the authorship inclusions/changes. Following is the order of the authors/co-authors as appear in the manuscript.
>
> "NOX activation in reactive astrocytes regulates astrocytic LCN2 expression and neurodegeneration"
>
> Ruijia Liu1, 2, Jun Wang2, Yang Chen2, Jenelle M. Collier2, 3, Okan Capuk2, Shijie Jin4, Ming Sun5, Sujan K. Mondal6, Theresa L. Whiteside6, Donna B. Stolz5, Yongjie Yang4, Gulnaz Begum2*
>
> Please reply to this email "ASAP"
>
> Thank you,
> Gulnaz Begum

--
//

Shijie Jin, Ph.D. (金 世杰 , 김 세걸, キン セイケツ)
Research associate
Department of Neuroscience Yang Lab
Tufts University School of Medicine
136 Harrison Ave.
Arnold 711
Boston MA, 02111

e-mail: jin.shijie1977@gmail.com
Shijie.Jin@tufts.edu
jin_shijie@hotmail.com
Phone: (617) 372-0256

Re: Author agreement_CDDIS-21-4158RR

Sun, Ming <mis23@pitt.edu>
Fri 4/1/2022 9:08 AM

To: Begum, Gulnaz <gub6@pitt.edu>

Congratulations for the acceptation. Yes, I agree to the authorship inclusions/changes.

Thanks

From: Begum, Gulnaz <gub6@pitt.edu>
Sent: Thursday, March 31, 2022 7:37 PM
To: Sun, Ming
Subject: Author agreement_CDDIS-21-4158RR

Dear Ming,

Our manuscript entitled "NOX activation in reactive astrocytes regulates astrocytic LCN2 expression and neurodegeneration" has been accepted for publication in Cell death and disease journal. Please reply to this email stating you agree to the authorship inclusions/changes. Following is the order of the authors/co-authors as appear in the manuscript.

NOX activation in reactive astrocytes regulates astrocytic LCN2 expression and neurodegeneration

Ruijia Liu1, 2, Jun Wang2, Yang Chen2, Jenelle M. Collier2, 3, Okan Capuk2, Shijie Jin4, Ming Sun5, Sujan K. Mondal6, Theresa L. Whiteside6, Donna B. Stolz5, Yongjie Yang4, Gulnaz Begum2*

Please reply to this email "ASAP"

Thank you,
Gulnaz Begum

Re: Author agreement_CDDIS-21-4158RR

Mondal, Sujan Kumar <mondals@upmc.edu>
Fri 4/1/2022 10:59 AM
To: Begum, Gulnaz <gub6@pitt.edu>

Dear Gulnaz,

I agree to the authorship inclusions/changes.

Thanks,
Sujan

From: Begum, Gulnaz <gub6@pitt.edu>
Sent: Friday, April 1, 2022 9:55 AM
To: Mondal, Sujan Kumar <SUM81@pitt.edu>
Subject: Author agreement_CDDIS-21-4158RR

Dear Sujan,

Our manuscript entitled "NOX activation in reactive astrocytes regulates astrocytic LCN2 expression and neurodegeneration" has been accepted for publication in Cell death and disease journal. There are following changes in the authorship: "Yang Chen"; "Okan Capuk"; "Ming Sun", and "Jenelle M Collier" have been included because they collected new experimental results in the re submission. Dandan Sun has been removed from the authorship based on her request. Please reply to this email stating you agree to the authorship inclusions/changes. Following is the order of the authors/co-authors as appear in the manuscript.

"NOX activation in reactive astrocytes regulates astrocytic LCN2 expression and neurodegeneration"

Ruijia Liu1, 2, Jun Wang2, Yang Chen2, Jenelle M. Collier2, 3, Okan Capuk2, Shijie Jin4, Ming Sun5, Sujan K. Mondal6, Theresa L. Whiteside6, Donna B. Stolz5, Yongjie Yang4, Gulnaz Begum2*

Please reply to this email "**ASAP**"

Thank you,
Gulnaz Begum

RE: Author agreement_CDDIS-21-4158RR

Whiteside, Theresa <whitesidetl@upmc.edu>
Fri 4/1/2022 9:53 AM

To: Begum, Gulnaz <gub6@pitt.edu>

Hi! This is to confirm that I agree to the authorship inclusions/changes in the manuscript referred to above, TW

Theresa L. Whiteside, PhD, MDHC
Professor of Pathology, Immunology and Otolaryngology
UPMC Hillman Cancer Center
University of Pittsburgh Cancer Institute
5117 Centre Avenue
Pittsburgh, PA 15213
Phone (412)624-0096

From: Begum, Gulnaz <gub6@pitt.edu>
Sent: Thursday, March 31, 2022 8:45 PM
To: Whiteside, Theresa <TWHITE@pitt.edu>
Cc: Whiteside, Theresa <whitesidetl@upmc.edu>
Subject: Author agreement_CDDIS-21-4158RR

Dear Dr. Theresa,

Our manuscript entitled "NOX activation in reactive astrocytes regulates astrocytic LCN2 expression and neurodegeneration" has been accepted for publication in Cell death and disease journal. Please reply to this email stating you agree to the authorship inclusions/changes. Following is the order of the authors/co-authors as appear in the manuscript.

NOX activation in reactive astrocytes regulates astrocytic LCN2 expression and neurodegeneration

Ruijia Liu[1, 2], Jun Wang[2], Yang Chen[2], Jenelle M. Collier[2, 3], Okan Capuk[2], Shijie Jin[4], Ming Sun[5], Sujan K. Mondal[6], Theresa L. Whiteside[6], Donna B. Stolz[5], Yongjie Yang[4], Gulnaz Begum[2*]

Please reply to this email **"ASAP"**

Thank you,
Gulnaz Begum

Re: Author agreement_CDDIS-21-4158RR

Stolz, Donna B <donna.stolz@pitt.edu>
Fri 4/1/2022 10:01 AM
To: Begum, Gulnaz <gub6@pitt.edu>

I agree with the authorship changes.
Donna Stolz

Sent from my iPhone 11
Please excuse typos!

> On Apr 1, 2022, at 9:41 AM, Begum, Gulnaz <gub6@pitt.edu> wrote:
>
> Dear Donna,
>
> Our manuscript entitled "NOX activation in reactive astrocytes regulates astrocytic LCN2 expression and neurodegeneration" has been accepted for publication in Cell death and disease journal. There are following changes in the authorship: Yang Chen; Okan Capuk; Ming Sun, and Jenelle M Collier have been included because they collected new experimental results in the re submission. Dandan Sun has been removed from the authorship based on her request. Please reply to this email stating you agree to the authorship inclusions/changes. Following is the order of the authors/co-authors as appear in the manuscript.
>
> "NOX activation in reactive astrocytes regulates astrocytic LCN2 expression and neurodegeneration"
>
> Ruijia Liu1, 2, Jun Wang2, Yang Chen2, Jenelle M. Collier2, 3, Okan Capuk2, Shijie Jin4, Ming Sun5, Sujan K. Mondal6, Theresa L. Whiteside6, Donna B. Stolz5, Yongjie Yang4, Gulnaz Begum2*
>
> Please reply to this email "ASAP"
>
> Thank you,
> Gulnaz Begum

Re: Author agreement_CDDIS-21-4158RR

Yang, Yongjie <Yongjie.Yang@tufts.edu>
Thu 3/31/2022 8:56 PM
To: Begum, Gulnaz <gub6@pitt.edu>

Yes.

On Mar 31, 2022, at 8:47 PM, Begum, Gulnaz <gub6@pitt.edu> wrote:

Dear Dr. Yongjie,

Our manuscript entitled "NOX activation in reactive astrocytes regulates astrocytic LCN2 expression and neurodegeneration" has been accepted for publication in Cell death and disease journal. Please reply to this email stating you agree to the authorship inclusions/changes. Following is the order of the authors/co-authors as appear in the manuscript.

NOX activation in reactive astrocytes regulates astrocytic LCN2 expression and neurodegeneration

Ruijia Liu[1, 2], Jun Wang[2], Yang Chen[2], Jenelle M. Collier[2, 3], Okan Capuk[2], Shijie Jin[4], Ming Sun[5], Sujan K. Mondal[6], Theresa L. Whiteside[6], Donna B. Stolz[5], Yongjie Yang[4], Gulnaz Begum[2*]

Please reply to this email **"ASAP"**

Thank you,
Gulnaz Begum

RE: Author agreement_CDDIS-21-4158RR

Sun, Dandan <sund@upmc.edu>
Fri 4/1/2022 10:18 AM
To: Begum, Gulnaz <gub6@pitt.edu>

Dear Dr. Begum:
Congratulations on your new study! Yes, as we discussed, this is your new, independent research project, I provided some consultant in the study's development, but I decided to remove my name from the authorship.

Thanks.
Dandan

===================================
Dandan Sun, MD., PhD, FAHA
Professor, Dept. of Neurology
Endowed Chair, Brain Disorders Research
Pittsburgh Institute For Neurodegenerative Diseases
Univ. of Pittsburgh
VA Research Career Scientist
7016 Biomedical Science Tower-3 (BST-3)
3501 Fifth Ave.
Pittsburgh, PA 15260, USA
Phone: 412-624-0418
Fax: 412-648-3321
e-mail: sund@upmc.edu
http://www.neurology.upmc.edu/faculty/Sun_Dandan.html
===================================

From: Begum, Gulnaz <gub6@pitt.edu>
Sent: Thursday, March 31, 2022 8:56 PM
To: Sun, Dandan <sund@upmc.edu>
Subject: Author agreement_CDDIS-21-4158RR

Dear Dr. Sun,

Our manuscript entitled "NOX activation in reactive astrocytes regulates astrocytic LCN2 expression and neurodegeneration" has been accepted for publication in "Cell death and disease" journal.
As per your request, I have removed your name from the manuscript. Please reply to this email stating you agree to the authorship changes. Following is the order of the authors/co-authors as appear in the manuscript.

NOX activation in reactive astrocytes regulates astrocytic LCN2 expression and neurodegeneration

Ruijia Liu[1, 2], Jun Wang[2], Yang Chen[2], Jenelle M. Collier[2, 3], Okan Capuk[2], Shijie Jin[4], Ming Sun[5], Sujan K. Mondal[6], Theresa L. Whiteside[6], Donna B. Stolz[5], Yongjie Yang[4], Gulnaz Begum[2*]

Please reply to this email **"ASAP"**

Thank you,
Gulnaz Begum
